# Combined Omics Analysis Further Unveils the Specific Role of Butyrate in Promoting Growth in Early-Weaning Animals

**DOI:** 10.3390/ijms24021787

**Published:** 2023-01-16

**Authors:** Bin Zhang, Mengqi Liu, Zhengkai Yue, Xiaoyang Chen, Chenyang Li, Lei Liu, Fuchang Li

**Affiliations:** 1 Key Laboratory of Efficient Utilization of Non-grain Feed Resources (Co-construction by Ministry and Province), Ministry of Agriculture and Rural Affairs, Shandong Provincial Key Laboratory of Animal Biotechnology and Disease Control and Prevention, Department of Animal Science, Shandong Agricultural University, Taian 271018, China; 2 State Key Laboratory of Animal Nutrition, Institute of Animal Science, Chinese Academy of Agricultural Sciences, Beijing 100193, China; 3 College of Animal Science and Technology, Northwest A&F University, Xi'an 712100, China; 4 College of Animal Science and Technology, China Agricultural University, Beijing 100083, China

**Keywords:** dietary butyrate, early weaning, intestinal epithelial barrier, microbiome–metabolome, inflammation and appetite, microbe–gut–brain axis

## Abstract

Abnormal mutations in the microbial structure of early-weaning mammals are an important cause of enteritis. Based on the multiple known beneficial functions of butyrate, we hypothesized that butyrate would alleviate the imbalance of intestinal homeostasis induced by early weaning in animals. However, the mechanisms of action between butyrate and intestinal microbes are still poorly explored. In this study, we aimed to investigate whether butyrate exerts beneficial effects on the structure of the intestinal flora of weanling rabbits and their intestinal homeostasis, growth and development, and we attempted to elucidate the potential mechanisms of action through a combined omics analysis. We found that dietary butyrate upregulated the transcription of tight junction-related proteins in the epithelial barrier and improved the intestinal microbial structure by suppressing harmful bacteria and promoting beneficial ones. Intestinal and plasma metabolomes were also altered. The bile acid secretion, α-linolenic acid, apoptotic, and prostate cancer pathways responded to the positive dietary butyrate-induced metabolic changes in the weanling rabbits, resulting in the inhibition of inflammation, improved antioxidant capacity, increased rates of cell proliferation and survival, and decreased levels of apoptosis. Additionally, dietary butyrate suppressed the release of pro-inflammatory factors and enhanced positive appetite regulation, which increased the average daily gain of the rabbits. These results demonstrated that dietary butyrate can help maintain the integrity of the intestinal epithelial barrier, improve the structural composition of the intestinal microflora, enhance organismal metabolism, inhibit inflammation, reduce post-weaning anorexia, and promote growth and development in early-weaning rabbits. These positive effects of dietary butyrate were exerted via the modulation of the microbe–gut–brain axis.

## 1. Introduction

In modern, large-scale, intensive animal production, early weaning is used to improve economic efficiency, resulting in greater early-weaning pressure. The associated stressors lead to the loss of appetite, growth retardation, a high incidence of diarrhea, and increased risk of disease and mortality [1,2]. These manifestations are usually caused by intestinal barrier dysfunction and are characterized by pathological alterations in intestinal morphology, tight junction damage, and inflammation [3,4]. The use of antibiotics has been a common response to early-weaning-related damage for decades; however, owing to the increases in antibiotic resistance and antibiotic accumulation in food, antibiotics are now strictly regulated or even banned in modern animal production [5,6]. These observations highlight the urgent need to explore alternative non-antibiotic strategies.

The small intestine is the main site of nutrient digestion and absorption, as well as an important defense barrier against harmful exogenous and endogenous substances or pathogens [7]. Gastrointestinal barriers consist of multilayered systems comprising defense mechanisms provided by the intestinal epithelium, as well as immune and enteric nervous system components [8]. The epithelial barrier, which includes intestinal epithelial cells and intercellular junctions, especially tight junctions, plays a central role in host defenses against invasion by microbiota, toxins, and antigens from the exogenous environment [9]. In both diarrheal and inflammatory bowel diseases, the barrier function of the intestinal epithelium can be impaired [10]. Post-weaning diarrhea is a multifactorial gastrointestinal disease, with malnutrition as one of its main causes [11,12]. The gastrointestinal tract is a complex, balanced ecosystem, and the intestinal epithelial barrier is a dynamic, physical barrier that continuously interacts with the intestinal microbiota [13,14]. It has been reported that the gut microbiota influence the maintenance of the intestinal epithelial barrier by modulating the immune system [15].

The gut microbiome in healthy individuals is a balanced community of a multitude of microorganisms, including bacteria, viruses, phages, archaea, and fungi [16]. The bacterial community participates in the maintenance of intestinal homeostasis through interactions with the immune system, thereby inhibiting the growth of pathogens [17]. Additionally, the gut microbiome mediates the regulation of the intestinal inflammatory response [18]. Importantly, certain microorganisms in the gut can digest dietary fiber, producing metabolites that have a positive effect on the intestinal epithelial barrier, including short-chain fatty acids [19] such as butyrate, which is essential for the maintenance of intestinal health. Butyrate not only serves as a source of energy for intestinal epithelial cells and helps maintain the integrity of the intestinal mucosa but also prevents cellular inflammation and promotes the removal of dysfunctional cells [20,21]. Ultimately, butyrate helps maintain intestinal homeostasis through its anti-inflammatory effects [22].

Numerous studies have shown that dietary butyrate is effective at promoting intestinal development and organismal growth in weaning mammals [23,24,25]. Although butyrate is known to have numerous beneficial effects on the host, including on the gut, there is growing evidence that butyrate can exert beneficial effects on neurocentral regulation via the microbial–gut–brain axis [26]. To date, however, little is known about the mechanisms by which butyrate influences the intestinal microflora and regulates which flora in the gut improve the intestinal environment, thereby altering the body’s metabolism and dialogue between the gut microbiota and the central nervous system to promote healthy growth.

In this study, we investigated the effect of dietary butyrate on the growth status of early-weaning rabbits (30 days old). For this, we undertook a comprehensive analysis of the effect of dietary butyrate on the microstructure of the ileal epithelium, the transcriptional levels of physical intestinal epithelium barrier regulators, the ileum microbiome, the ileum metabolome, the plasma metabolome, inflammatory responses, and appetite regulation. Our findings provide a comprehensive description of the mechanism underlying the effect of dietary butyrate on the growth of early-weaning rabbits, as well as a reference for future research on and applications of butyrate in animal health care.

## 2. Results

### 2.1. Effects of Dietary Butyrate on the Growth Status of Rabbits

**Figure 1:**
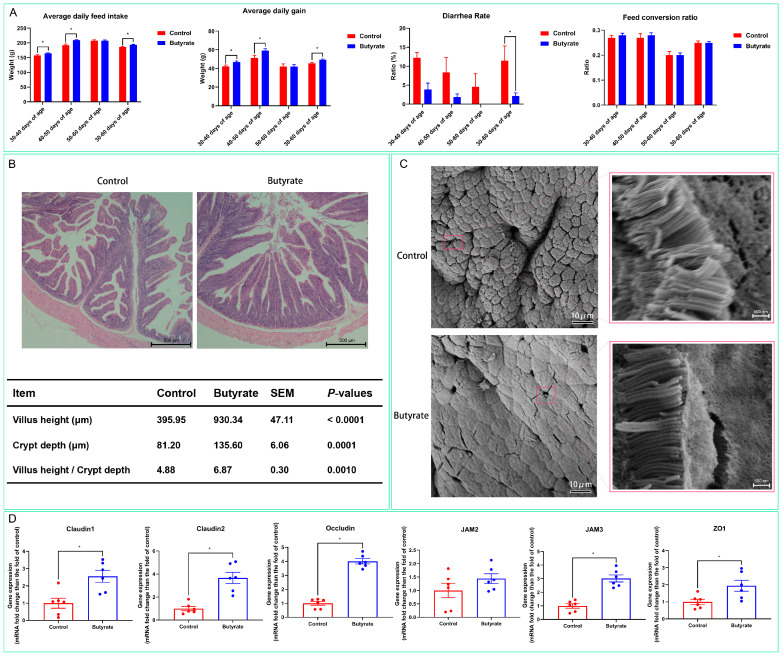
Effect of dietary butyrate on growth status and intestinal epithelial barrier in weanling rabbits. (**A**) Growth of weanling rabbits from 30 days of age to 60 days of age (*n* = 60). (**B**) Observation of ileum villus height and crypt depth in weanling rabbits at 50 days of age (*n* = 6). (**C**) Observation of the microstructure of the ileum mucosal epithelium in weanling rabbits at 50 days of age (*n* = 6). (**D**) Expression of genes associated with the intestinal epithelial barrier in the ileal mucosal epithelium of weanling rabbits at 50 days of age (*n* = 6). Date are expressed as mean ± SEM, * *p* < 0.05.

We analyzed rabbit growth at different growth periods and found that dietary butyrate significantly increased the average daily feed intake and average daily gain of the animals at 30–40 days of age, 40–50 days of age, and 30–60 days of age (*p* < 0.05) (Figure 1A). Meanwhile, the incidence of diarrhea was significantly reduced in the butyrate group at 30–60 days of age (*p* < 0.05); however, dietary butyrate did not significantly affect the feed conversion ratio (*p* > 0.05). The analysis of the H&E-stained sections of the ileum (Figure 1B) indicated that dietary butyrate significantly increased the villus height and the villus height/crypt depth ratio (*p* < 0.05), while results of the scanning electron microscopic analysis of the ileal lumen showed that dietary butyrate significantly improved the structural integrity of the luminal epithelium of the ileum (Figure 1C). Furthermore, dietary butyrate significantly upregulated the expression of the ileal epithelial barrier-related genes claudin-1, claudin-2, occludin, junctional adhesion molecule 3 (*JAM3*), and zonula occludens 1 (*ZO-1*) (Figure 1D, *p* < 0.05), but it had no significant effect on that of *JAM2*, as determined with RT-qPCR (Figure 1D, *p* > 0.05).

### 2.2. Determination of Inflammation in Different Tissues and Organs

**Figure 2:**
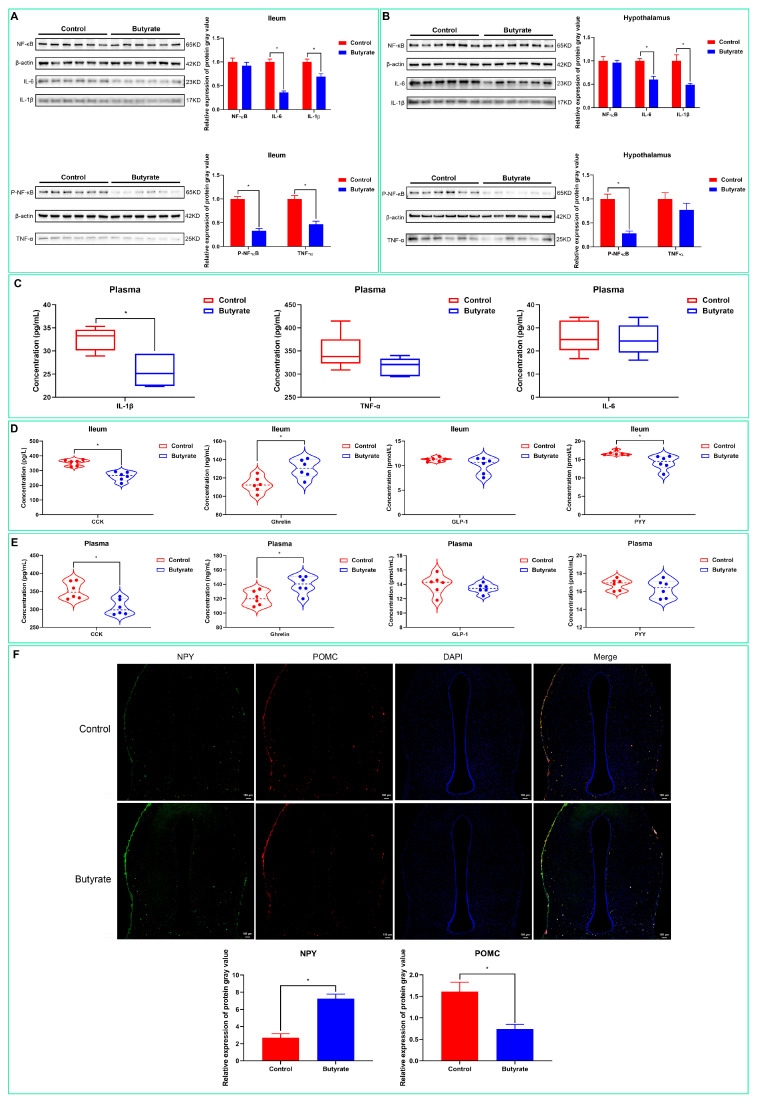
Effect of butyrate on the expression of inflammatory factors and appetite proteins in the ileum, plasma and hypothalamus of weanling rabbits. (**A**) Expression of inflammatory factors in ileal tissues. (**B**) Expression of inflammatory factors in hypothalamic tissue. (**C**) Expression of inflammatory factors in plasma. (**D**) Expression levels of appetite regulation related brain–gut hormones in the ileum. (**E**) Expression levels of brain–gut hormones associated with appetite regulation in plasma. (**F**) Expression levels of proteins associated with appetite regulation in the hypothalamus. Date are expressed as mean ± SEM, *n* = 6, * *p* < 0.05 (Western blots for each set of reference protein and target protein were from one blot, and the black line represents the cropped edge of the blot).

To investigate whether dietary butyrate could alleviate the inflammation caused by early weaning, we examined the levels of inflammatory factors in the ileum, hypothalamus, and plasma. As shown in Figure 2A, dietary butyrate significantly downregulated the protein levels of IL-1β, IL-6, p-NF-κB p65, and TNF-α in the ileum (*p* < 0.05) but had no significant effect on the protein expression of NF-κB p65 (*p* > 0.05). In the hypothalamus (Figure 2B), meanwhile, dietary butyrate significantly reduced the protein levels of IL-1β, IL-6, and p-NF-κB p65 (*p* < 0.05); however, no effect on the protein expression of NF-κB p65 and TNF-ɑ was observed (*p* > 0.05). An ELISA for the levels of inflammatory factors in the plasma revealed that dietary butyrate significantly downregulated the level of IL-1β in rabbit plasma (*p* < 0.05) but had a non-significant effect on that of TNF-α or IL-6 (*p* > 0.05). These results suggested that dietary butyrate alleviated early-weaning-induced inflammation to varying degrees and in different tissues.

### 2.3. Effects of Dietary Butyrate on Appetite Regulation in Early-Weaning Rabbits

ELISAs for the levels of appetite-regulating hormones demonstrated that dietary butyrate significantly decreased the levels of CCK and PYY and upregulated those of ghrelin in the mucosal surface of the ileal lumen (*p* < 0.05) (Figure 2D) In plasma, meanwhile, dietary butyrate significantly reduced the content of CCK and increased that of ghrelin (*p* < 0.05) (Figure 2E). To further confirm these results, we performed tissue immunofluorescence staining for appetite-related proteins in hypothalamic tissue. As shown in Figure 2F, dietary butyrate significantly upregulated the protein expression of NPY and downregulated that of POMC (*p* < 0.05).

### 2.4. The Effect of Dietary Butyrate on Intestinal Microbial Community Composition and Function in Early-Weaning Rabbits

**Figure 3:**
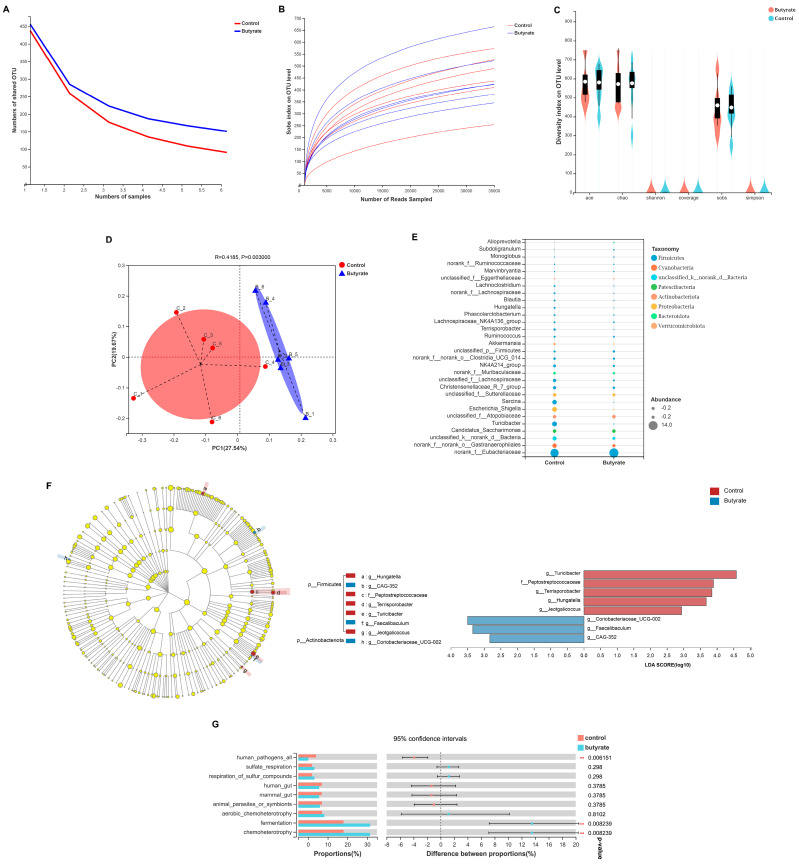
Differential analysis of species composition and functional prediction of gut microorganisms in the butyrate and control groups. (**A**) Pan/core species curve. (**B**) Rarefaction curves. (**C**) Alpha diversity. (**D**) Beta diversity analysis. (**E**) Microbial community composition analysis. (**F**) Lefse multilevel species difference discriminant analysis. (**G**) Wilcoxon rank-sum test on function. **: 0.001 < *p* ≤ 0.01.

To investigate the effects of dietary butyrate on the community structure of the ileal gut microbiota and microbial functions, we performed 16S rRNA gene sequencing. As shown in Figure 3A,B, the pan/core species and dilution curve analysis indicated that the sequencing sample size was sufficient and that the analysis results were valid. Furthermore, dietary butyrate had no significant effect on gut microbial α-diversity (*p* > 0.05) (Figure 3C). However, PCoA results at the gut microbial OTU level showed that dietary butyrate had a significant effect on β-diversity (Figure 3D, *p* < 0.05). The top 30 most abundant phyla at the genus level among the gut microorganisms were analyzed using species hierarchical clustering. As shown in Figure 3E, Firmicutes was the most abundant genus in both groups, and the proportion of Firmicutes was higher in the butyrate group than in the control group.

To determine the extent to which microorganisms contributed to the achievement of group differences between the two experimental groups, we performed a LEfSe multilevel species difference discriminant analysis. As shown in Figure 3F, a total of eight species were enriched at the genus level: five (g__Turicibacter, f__Peptostreptococcaceae, g__Terrisporobacter, g__Hungatella, and g__Jeotgalicoccus) in the control group and three (g__Coriobacteriaceae_UCG-002, g__Faecalibaculum, and g__CAG-352) in the butyrate group. Of the eight enriched species, seven belonged to Firmicutes and one belonged to Actinobacteriota. For the functional annotation of gut microbes, we matched the OTUs with the FAPROTAX database. As shown in Figure 3G, the pathogenic potential of intestinal microorganisms was significantly higher in the control group than in the butyrate group. Moreover, diet fermentability and the level of chemoenergetic heterotrophy were significantly greater in the butyrate group than in the control group (*p* < 0.05).

### 2.5. The Relationships among Intestinal Microorganisms and between Intestinal Microorganisms and Their Hosts

**Figure 4:**
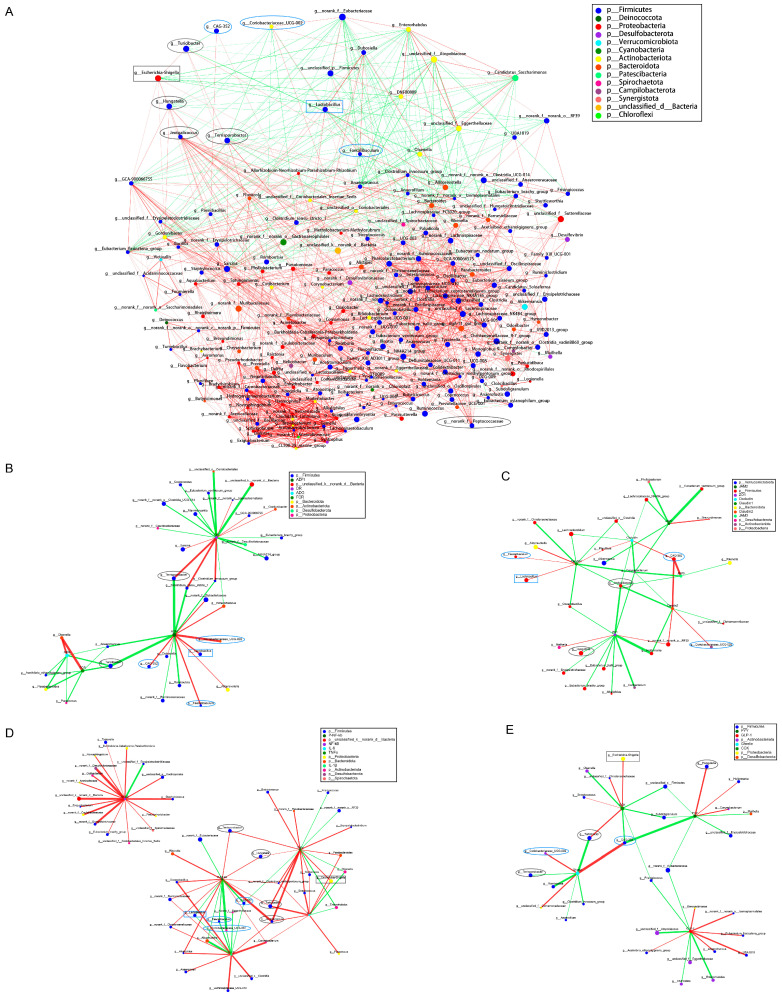
Correlation analysis between gut microbes and traits in weanling rabbits. (**A**) Network analysis between species in the microbiome of the ileum. (**B**) Microorganisms and growth status. (**C**) Microorganisms and the intestinal epithelial barrier. (**D**) Microorganisms and inflammatory factors. (**E**) Microorganisms and gut–brain hormones. The red line indicates a significant positive correlation, and the green line indicates a significant negative correlation (*p* < 0.05). In the black circles are the differentially dominant bacteria in the control group; in the blue circles are the differentially dominant bacteria in the butyrate group.

To study the interactions among the main microorganisms of the rabbit gut, we performed a one-factor, genus-level network analysis of the 208 species that were enriched in the gut. As shown in Figure 4A, a significant positive relationship was detected among the differentially dominant bacteria in each of the control and butyrate groups while a significant negative relationship was seen between these microorganisms in the two groups (*p* < 0.05). We also found a significant positive correlation between the differentially dominant bacteria within the control group and g_Escherichia–Shigella, while the differentially dominant bacteria within the butyrate group showed a significant positive correlation with g_Lactobacillus (*p* < 0.05).

We constructed a correlation network by calculating the correlation between the ileal microbiome at the genus level (all 208 species) and the growth status of the rabbits through a two-factor network analysis. As shown in Figure 4B, in the control group, significant negative correlations were found between the differentially dominant bacteria g__Terrisporobacter and g__Turicibacter and the average daily feed intake (*p* < 0.05), as well as between g__Turicibacter and the average daily gain and feed conversion rate (*p* < 0.05). Meanwhile, g__Terrisporobacter was significantly and positively correlated with the rate of diarrhea (*p* < 0.05). In the butyrate group, three differentially dominant bacteria (g__Coriobacteriaceae_UCG-002, g__Faecalibaculum, and g__CAG-352) and g_Lactobacillus were significantly and positively correlated with the average daily feed intake of the early-weaning rabbits (*p* < 0.05).

As shown in Figure 4C, a two-factor network analysis of gut microbes and the expression of gut epithelial barrier-related genes indicated that the differentially dominant bacteria g__Jeotgalicoccus, g__Terrisporobacter, g__Turicibacter, and g__Hungatella in the control group and g__Faecalibaculum, g__CAG-352, and g__Coriobacteriaceae_UCG-002 in the butyrate group, as well as g_Lactobacillus, influenced the expression of gut epithelial barrier-related genes. g__Jeotgalicoccus was significantly and negatively correlated with the expression of claudin-1, claudin-2, occludin, *JAM3*, and *ZO-1*, while g__Hungatella was significantly and negatively correlated with that of *ZO-1* (*p* < 0.05). In the butyrate group, significant positive correlations were found between the differentially dominant bacterium g__Faecalibaculum and claudin-1 gene expression, between g__Coriobacteriaceae_UCG-002 and claudin-2, and between g__CAG-352 and occludin, claudin-2, and *JAM3* (*p* < 0.05). Additionally, g_Lactobacillus was significantly and positively correlated with the gene expression of claudin-1 (*p* < 0.05).

Similarly, a two-factor network analysis of the relationship between ileal gut microorganisms and ileal tissue inflammatory factors was undertaken in both groups (Figure 4D). We found that the differentially dominant bacteria in the control group, such as g_Escherichia–Shigella, were significantly and positively correlated with intestinal inflammatory factors. Differentially dominant bacteria in the butyrate group, such as g_Lactobacillus, were significantly and negatively correlated with intestinal inflammatory factors. In the control group, the differentially dominant bacteria g__Terrisporobacter and g__Hungatella were significantly and positively correlated with TNF-α and p-NF-κB p65; g__Turicibacter was significantly and positively correlated with TNF-ɑ, IL-6, and p-NF-κB p65; and g__Jeotgalicoccus was significantly and positively correlated with TNF-ɑ, IL-6, IL-1β, and p-NF-κB p65 (*p* < 0.05). In the butyrate group, the differentially dominant bacteria g__Faecalibaculum and g__Coriobacteriaceae_UCG-002 were significantly and negatively correlated with IL-1β and p-NF-κB p65, while g__CAG-352 was significantly and negatively correlated with IL-1β, TNF-α, and P-NF-κB p65 (*p* < 0.05). Meanwhile, g_Escherichia–Shigella was significantly and positively correlated with the inflammatory factors TNF-α and IL-1β, and g_Lactobacillus was significantly and negatively correlated with IL-1β and p-NF-κB p65 (*p* < 0.05).

The results of the two-factor network analysis of the correlation between microorganisms and gut–brain hormones in the intestinal lumen epithelial mucosa are shown in Figure 4E. We found that, in the control group, the differentially dominant bacteria g__Turicibacter and g_Escherichia–Shigella were significantly and positively correlated with the gut–brain hormone CCK (both *p* < 0.05), while g__Hungatella was positively correlated with the gut–brain hormone PYY (*p* < 0.05). G__Turicibacter and g__Terrisporobacter were significantly and negatively correlated with the gut–brain hormone ghrelin (*p* < 0.05). In the butyrate group, the differentially dominant bacteria g__CAG-352 and g__Coriobacteriaceae_UCG-002 were significantly and positively correlated with ghrelin, whereas g__CAG-352 was significantly and negatively correlated with CCK and PPY (*p* < 0.05).

### 2.6. The Effect of Dietary Butyrate on the Ileum and Plasma Metabolism in Weanling Rabbits

**Figure 5:**
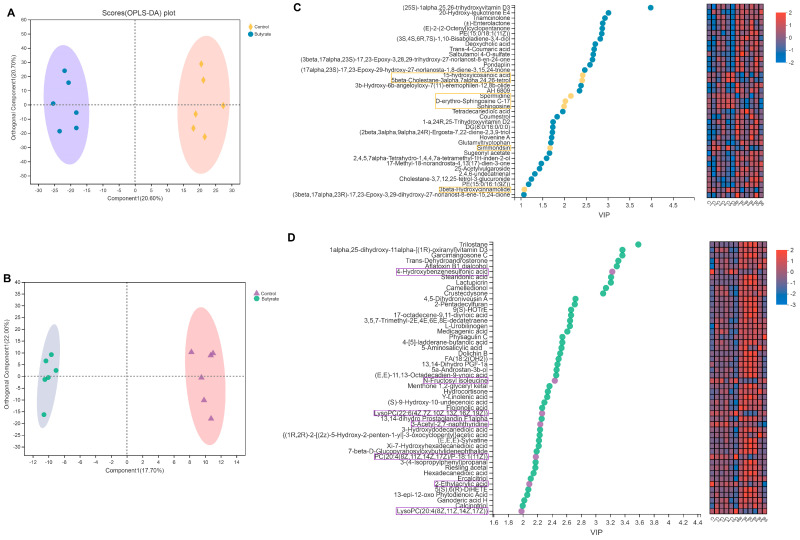
Differential analysis of metabolites between the control and butyrate groups in the ileum metabolome and plasma metabolome of weanling rabbits. (**A**) PLS–DA analysis of the ileal metabolome. (**B**) PLS–DA analysis of the plasma metabolome. (**C**) Analysis of the VIP values of the ileum metabolome. (**D**) Analysis of the VIP values of the plasma metabolome. VIP ≥ 1. The metabolites inside the yellow box in Figure C are the differentially dominant metabolites in the control group in the ileum; outside the yellow box are the differentially dominant metabolites in the butyrate group in the ileum. Metabolites inside the purple box in Figure D are the differentially dominant metabolites in the control group in plasma; outside the purple box are the differentially dominant metabolites in the butyrate group in plasma.

OPLS–DA results demonstrated that the ileal and plasma metabolite distributions were relatively independent between the control and butyrate groups, indicating that dietary butyrate exerted a relatively significant effect on the metabolism of the rabbits (Figure 5A,B). Additionally, based on the VIP scores obtained from the OPLS–DA, a complete hierarchical cluster analysis was performed for the metabolites associated with the top 50 VIP values using the Euclidean distance algorithm (Figure 5C,D). In the ileum, Seven dominant metabolites were found in the control group and 30 dominant metabolites were found in the butyrate group (Figure 5C, VIP ≥1, *p* < 0.05). In plasma, meanwhile, seven dominant metabolites were present in the control group and 43 dominant metabolites in the butyrate group (Figure 5D, VIP ≥1, *p* < 0.05).

### 2.7. Analysis of the Correlation of the Ileal Metabolome with Growth Status, Intestinal Epithelial Barrier Development, Intestinal Mucosal Inflammation, and Intestinal Hormone Levels in Early-Weaning Rabbits

**Figure 6:**
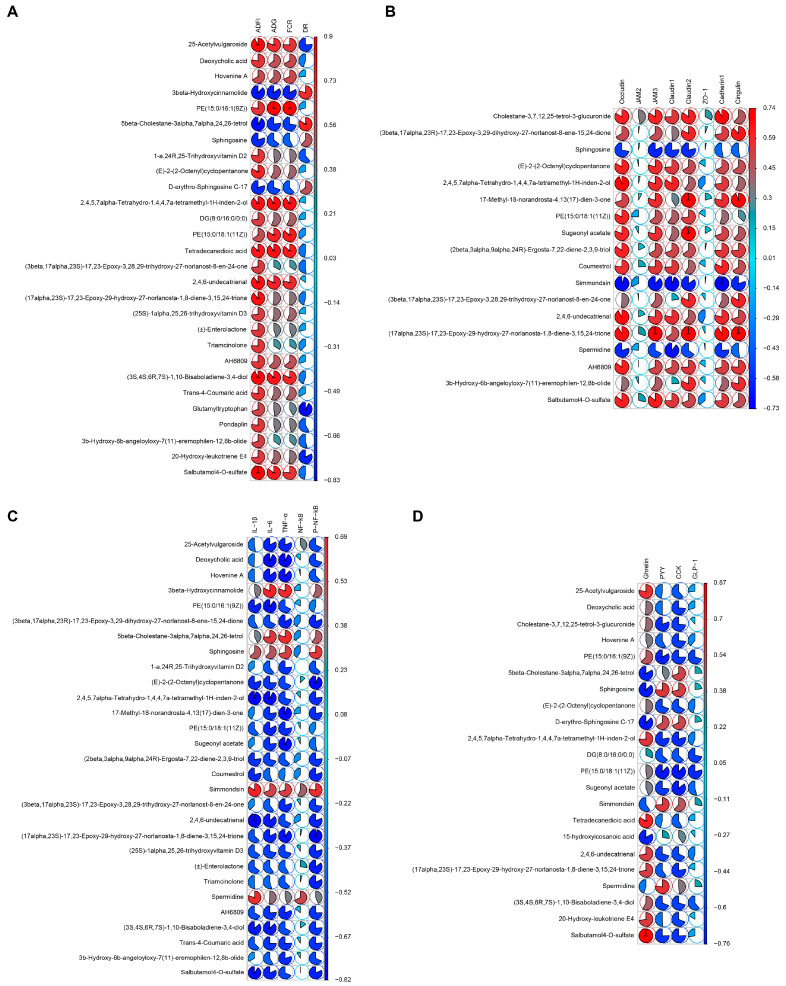
Correlation analysis of the ileal metabolome with growth status, intestinal epithelial barrier, inflammatory response and plasma hormone levels in weanling rabbits. (**A**) Ileum metabolome and growth status of weanling rabbits. (**B**) Ileal metabolome and intestinal epithelial barrier-related genes in weanling rabbits. (**C**) Ileal metabolome and intestinal inflammatory factors. (**D**) Ileal metabolome and gut–brain hormones in the intestine. *: 0.01 < *p* ≤ 0.05, **: 0.001 < *p* ≤ 0.01.

To determine the relationship of the ileal metabolome with growth status, intestinal epithelial barrier development, intestinal mucosal inflammation, and intestinal hormone levels in the rabbits, we undertook a Spearman’s correlation analysis of the association between the ileal metabolome and growth status, the expression of barrier-related genes in the ileum, the levels of intestinal inflammatory factors, and the levels of brain and intestinal hormones in the intestinal mucosa. The Benjamini and Hochberg method for controlling the FDR was used for *p*-value correction. As shown in Figure 6A, the metabolites that were significantly and positively correlated with the average daily feed intake, average daily weight gain, and feed conversion ratio included 25-acetylvulgaroside; deoxycholic acid; PE(15:0/16:1(9Z)); 2,4,5,7α-tetrahydro-1,4,4,7a-tetramethyl-1H-inden-2-ol; PE(15:0/18:1(11Z)); tetradecanedioic acid; 2,4,6-undecatrienal; (3S,4S,6R,7S)-1,10-bisaboladiene-3,4-diol; and salbutamol 4-O-sulfate. Those that were significantly and negatively correlated with the average daily feed intake, average daily weight gain, and feed conversion ratio included 3β-hydroxycinnamolide and 5β-cholestane-3α,7α,24,26-tetrol (R ≥ 0.6 or R ≤ −0.6, *p* < 0.05).

The analysis of the correlation between the intestinal metabolome and the expression of intestinal epithelial barrier-related genes led to the identification of metabolites that were significantly and positively correlated with intestinal epithelial barrier development. These mainly included cholestane-3,7,12,25-tetrol-3-glucuronide; 2,4,5,7α-tetrahydro-1,4,4,7a-tetramethyl-1H-inden-2-ol; 17-methyl-18-norandrosta-4,13(17)-dien-3-one; 2,4,6-undecatrienal; and (17α,23S)-17,23-epoxy-29-hydroxy-27-norlanosta-1,8-diene-3,15,24-trione (Figure 6B, R ≥ 0.6 or R ≤ −0.6, *p* < 0.05). Metabolites that were significantly and negatively associated with intestinal epithelial barrier development included sphingosine, simmondsin, and spermidine (Figure 6B, R ≥ 0.6 or R ≤ −0.6, *p* < 0.05).

We further assessed the putative correlations between the ileal metabolome and inflammatory factor expression in ileal tissue. The results showed that the metabolites 3β-hydroxycinnamolide; 5β-cholestane-3α,7α,24,26-tetrol; sphingosine; simmondsin; and spermidine were positively correlated with inflammatory factor expression (Figure 6C, R ≥ 0.6 or R ≤ −0.6, *p* < 0.05). The metabolites that were negatively correlated with inflammatory factor expression included 2,4,5,7α-tetrahydro-1,4,4,7a-tetramethyl-1H-inden-2-ol; (E)-2-(2-octenyl)cyclopentanone; (2β,3α,9α,24R)-ergosta-7,22-diene-2,3,9-triol; 2,4,6-undecatrienal; and (3S,4S,6R,7S)-1,10-bisaboladiene-3,4-diol (Figure 6C, R ≥ 0.6 or R ≤ −0.6, *p* < 0.05).

Finally, we undertook an analysis of the correlation between the ileal metabolome and appetite-related intestinal hormones. We found that the intestinal metabolites salbutamol 4-O-sulfate; tetradecanedioic acid; 25-acetylvulgaroside; 2,4,5,7α-tetrahydro-1,4,4,7a-tetramethyl-1H-inden-2-ol; and 2,4,6-undecatrienal, among others, were significantly and positively correlated with the intestinal hormone ghrelin (Figure 6D, R ≥ 0.6 or R ≤ −0.6, *p* < 0.05), whereas 5β-cholestane-3α,7α,24,26-tetrol; sphingosine; D-erythro-sphingosine C-17; and 15-hydroxyicosanoic acid were significantly and negatively correlated with ghrelin (Figure 6D, R ≥ 0.6 or R ≤ −0.6, *p* < 0.05). The intestinal metabolites that showed a significant positive correlation with PYY included sphingosine, simmondsin, and spermidine, while those that were negatively correlated with PYY included PE(15:0/16:1(9Z)); PE(15:0/18:1(11Z)); cholestane-3,7,12,25-tetrol-3-glucuronide; (E)-2-(2-octenyl)cyclopentanone; and 2,4,5,7α-tetrahydro-1,4,4,7a-tetramethyl-1H-inden-2-ol (Figure 6D, R ≥ 0.6 or R ≤ −0.6, *p* < 0.05). The gut metabolites that were significantly and positively correlated with CCK included 5β-cholestane-3α,7α,24,26-tetrol; sphingosine; and D-erythro-sphingosine C-17; the metabolites that were significantly and negatively correlated with CCK included PE(15:0/18:1(11Z)); sugeonyl acetate; salbutamol 4-O-sulfate; (17α,23S)-17,23-epoxy-29-hydroxy-27-norlanosta-1,8-diene-3,15,24-trione; and 2,4,5,7α-tetrahydro-1,4,4,7a-tetramethyl-1H-inden-2-ol (Figure 6D, R ≥ 0.6 or R ≤ −0.6, *p* < 0.05). The gut metabolites PE(15:0/16:1(9Z)), DG(8:0/16:0/0:0), and PE(15:0/18:1(11Z)) were significantly and negatively correlated with the gut hormone GLP-1 (Figure 6D, R ≥ 0.6 or R ≤ −0.6, *p* < 0.05).

### 2.8. Analysis of the Correlation between the Plasma Metabolome and Growth, Appetite Response, and Inflammation in Early-Weaning Rabbits

**Figure 7:**
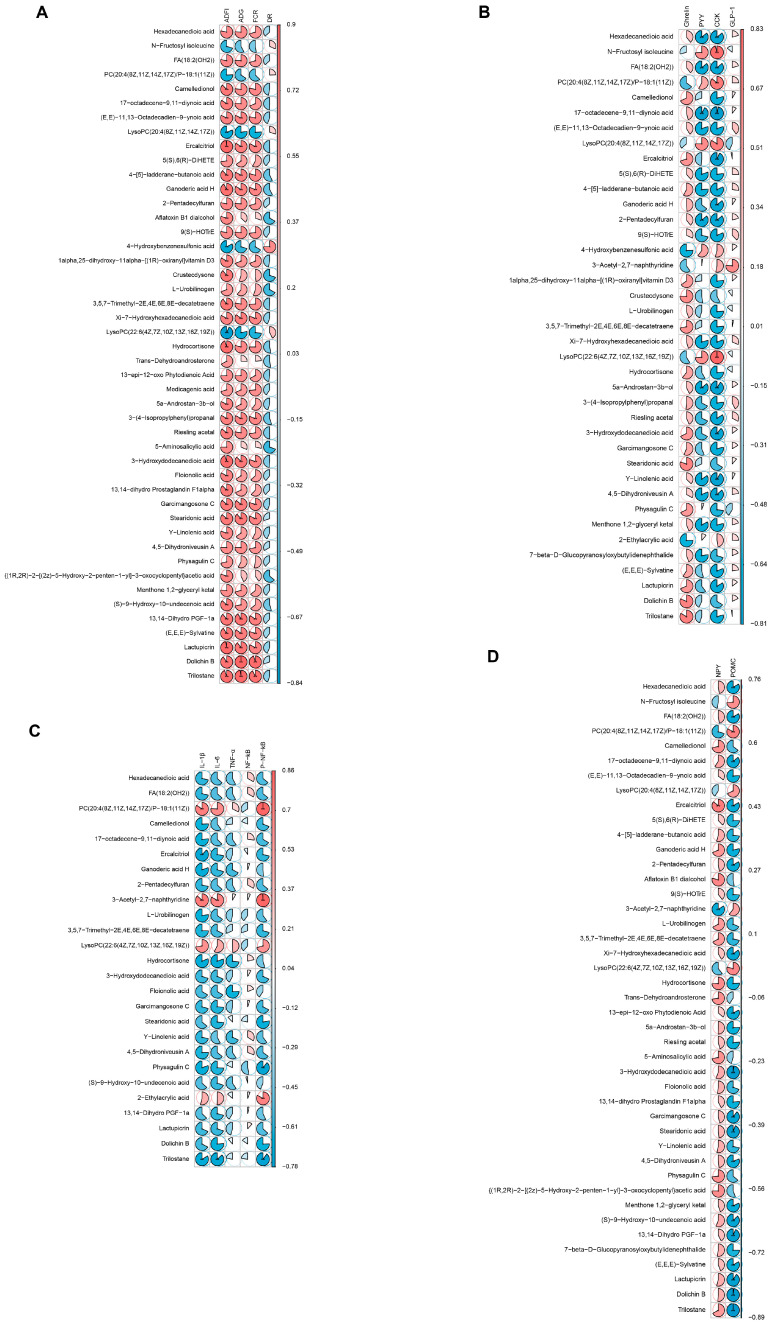
Correlation analysis of the plasma metabolome with growth status, plasma gut–brain hormones, inflammatory factors in the hypothalamus, and appetite-regulating proteins in the hypothalamus in weanling rabbits. *: 0.01 < *p* ≤ 0.05, **: 0.001 < *p* ≤ 0.01.

A Spearman’s correlation analysis was performed to investigate the relationship between the plasma metabolome and the growth status of the rabbits. As shown in Figure 7A, the main plasma metabolites that were significantly and positively correlated with the average daily feed intake, average daily weight gain, and feed conversion ratio in the rabbits were trilostane; dolichin B; lactupicrin; (E,E,E)-sylvatine; and 13,14-dihydro prostaglandin F1α (R ≥ 0.6 or R ≤ −0.6, *p* < 0.05). The main plasma metabolites found to be significantly and negatively correlated with the average daily feed intake, average daily weight gain, and feed conversion ratio were lysoPC(20:4(8Z,11Z,14Z,17Z)), 4-hydroxybenzenesulfonic acid, and lysoPC(22:6(4Z,7Z,10Z,13Z,16Z,19Z)) (R ≥ 0.6 or R ≤ −0.6, *p* < 0.05). Furthermore, 4-hydroxybenzenesulfonic acid was significantly and positively correlated with the rate of diarrhea in the rabbits, while aflatoxin B1 dialcohol, crustecdysone, L-urobilinogen, and 5-aminosalicylic acid showed a significant negative correlation with this parameter (R ≥ 0.6 or R ≤ −0.6, *p* < 0.05).

Regarding the association between gut–brain hormones and plasma metabolites, we found that the hormone ghrelin was significantly and positively correlated with the metabolites crustecdysone, stearidonic acid, dolichin B, trilostane, and ercalcitriol, as well as others, and it was significantly and negatively correlated with the metabolites 4-hydroxybenzenesulfonic acid and 2-ethylacrylic acid (Figure 7B, R ≥ 0.6 or R ≤ −0.6, *p* < 0.05). Meanwhile, the plasma metabolites that were significantly and positively correlated with PYY and CCK included N-fructosyl isoleucine, lysoPC(20:4(8Z,11Z,14Z,17Z), and lysoPC(22:6(4Z,7Z,10Z,13Z,16Z,19Z)), and those that were significantly and negatively correlated with PYY and CCK included hexadecanedioic acid; FA(18:2(OH2)); 17-octadecene-9,11-diynoic acid; 2-pentadecylfuran; and 5a-androstan-3b-ol (Figure 7B, R ≥ 0.6 or R ≤ −0.6, *p* < 0.05).

Next, we performed a correlation analysis to determine the relationship between the plasma metabolome and hypothalamic inflammation or the expression of hypothalamic appetite response-related proteins. For hypothalamic inflammation, the results showed that the plasma metabolites PC(20:4(8Z,11Z,14Z,17Z)/P-18:1(11Z)); 3-acetyl-2,7-naphthyridine; lysoPC(22:6(4Z,7Z,10Z,13Z,16Z,19Z)); and 2-ethylacrylic acid were significantly and positively correlated with inflammatory factor expression in the hypothalamus (R ≥ 0.6 or R ≤ −0.6, *p* < 0.05); those that were significantly and negatively correlated with inflammatory factor expression in the hypothalamus included trilostane, physagulin C, ercalcitriol, hydrocortisone, and dolichin B (R ≥ 0.6 or R ≤ −0.6, *p* < 0.05) (Figure 7C). Regarding hypothalamic appetite regulatory protein expression, as shown in Figure 7D, the plasma metabolites that were significantly and positively correlated with NPY protein expression in the hypothalamus included ercalcitriol, aflatoxin B1 dialcohol, physagulin C, hydrocortisone, and trilostane, while those that were significantly and negatively correlated with NPY protein expression in the hypothalamus included 3-acetyl-2,7-naphthyridine and PC(20:4(8Z,11Z,14Z,17Z)/P−18:1(11Z)) (R ≥ 0.6 or R ≤ −0.6, *p* < 0.05). We further found that the plasma metabolites that showed a significant positive correlation with POMC protein expression in the hypothalamus were PC(20:4(8Z,11Z,14Z,17Z)/P-18:1(11Z)), N-fructosyl isoleucine, lysoPC(22:6(4Z,7Z,10Z,13Z,16Z,19Z)), and lysoPC(20:4(8Z,11Z,14Z,17Z)), while those that exhibited a significant negative correlation with POMC protein expression in the hypothalamus included trilostane, dolichin B, lactupicrin, stearidonic acid, and 3-hydroxydodecanedioic acid (R ≥ 0.6 or R ≤ −0.6, *p* < 0.05).

### 2.9. Analysis of the Correlation between the Microbiome and the Metabolome of the Ileum

**Figure 8:**
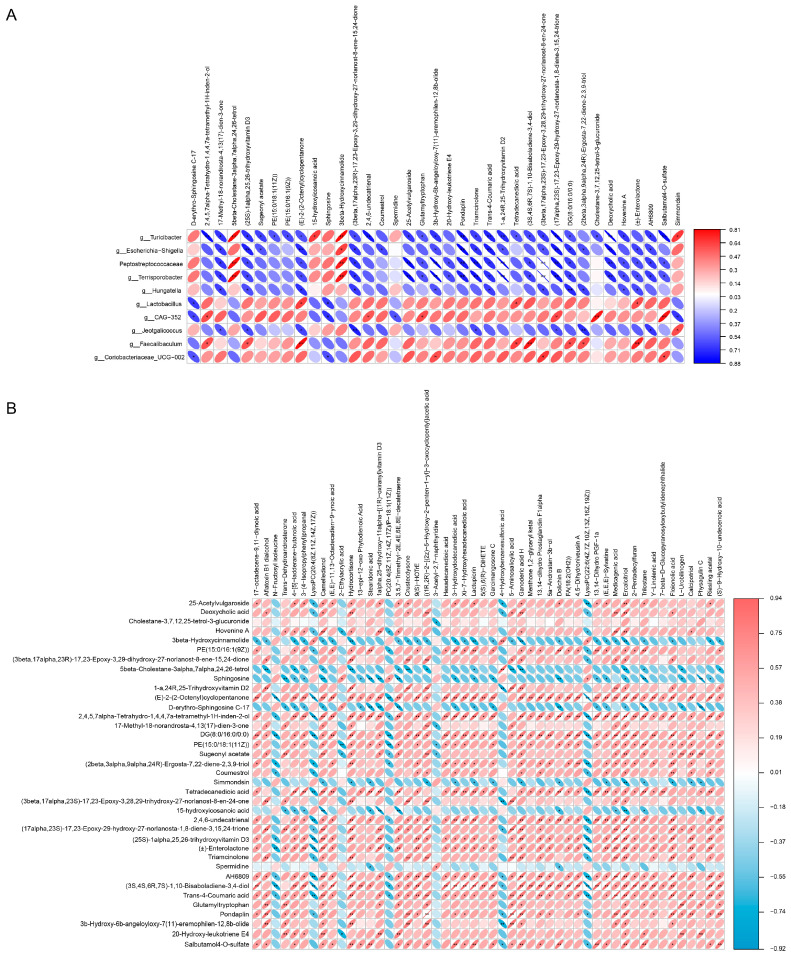
Correlation analysis between the gut microbiome and the gut metabolome and between the gut metabolome and the plasma metabolome. (**A**) Correlation analysis between the ileal microbiome and the ileal metabolome. (**B**) Correlation analysis between the intestinal metabolome and the plasma metabolome. *: 0.01 < *p* ≤ 0.05, **: 0.001 < *p* ≤ 0.01.

To determine the relationship of the ileal metabolome with the differentially dominant microorganisms in the control and butyrate groups, as well as with g_Escherichia–Shigella and g_Lactobacillus, we performed a Spearman’s correlation analysis (Figure 8A). The results indicated that the gut microbes could be divided into two main functional groups based on the correlation between them and the intestinal metabolites. The first microbial functional subgroup included g__Turicibacter, f__Peptostreptococcaceae, g__Terrisporobacter, g__Hungatella, g__Jeotgalicoccus, and g_Escherichia–Shigella, and the second comprised g__Coriobacteriaceae_UCG-002, g__Faecalibaculum, g__CAG-352, and g__Lactobacillus.

In the first microbial functional community, g__Turicibacter was significantly and positively correlated with 5β-cholestane-3α,7α,24,26-tetrol; 15-hydroxyicosanoic acid; 3β-hydroxycinnamolide; and simmondsin; it was negatively correlated with deoxycholic acid; 2,4,6-undecatrienal; tetradecanedioic acid; sugeonyl acetate; and salbutamol 4-O-sulfate, among other metabolites (R ≥ 0.6 or R ≤ −0.6, *p* < 0.05). Members of the Peptostreptococcaceae family were significantly and positively correlated with the metabolites 3β-hydroxycinnamolide and 5β-cholestane-3α,7α,24,26-tetrol and negatively correlated with deoxycholic acid, 25-acetylvulgaroside, salbutamol 4-O-sulfate, DG(8:0/16:0/0:0), and (3S,4S,6R,7S)-1,10-bisaboladiene-3,4-diol (R ≥ 0.6 or R ≤ −0.6, *p* < 0.05). The genus Terrisporobacter showed a significant positive correlation with the metabolites 3β-hydroxycinnamolide and 5β-cholestane-3α,7α,24,26-tetrol and a negative correlation with deoxycholic acid, salbutamol 4-O-sulfate, (E)-2-(2-octenyl)cyclopentanone, DG(8:0/16:0/0:0), 25-acetylvulgaroside, and other metabolites (R ≥ 0.6 or R ≤ −0.6, *p* < 0.05). The genus Hungatella was significantly and negatively correlated with, among other metabolites, DG(8:0/16:0/0:0); (E)-2-(2-octenyl)cyclopentanone; 17-methyl-18-norandrosta-4,13(17)-dien-3-one; (25S)-1α,25,26-trihydroxyvitamin D3; and (2β,3α,9α,24R)-ergosta-7,22-diene-2,3,9-triol (R ≥ 0.6 or R ≤ −0.6, *p* < 0.05). g__Jeotgalicoccus was significantly and positively correlated with simmondsin and negatively correlated with (E)-2-(2-octenyl)cyclopentanone; (2β,3α,9α,24R)-ergosta-7,22-diene-2,3,9-triol; (25S)-1α,25,26-trihydroxyvitamin D3; 17-methyl-18-norandrosta-4,13(17)-dien-3-one; and (17α,23S)-17,23-epoxy-29-hydroxy-27-norlanosta-1,8-diene-3,15,24-trione, among other metabolites (R ≥ 0.6 or R ≤ −0.6, *p* < 0.05). g_Escherichia–Shigella was significantly and positively correlated with 3β-hydroxycinnamolide and negatively correlated with the metabolites (E)-2-(2-octenyl)cyclopentanone; deoxycholic acid; (17α,23S)-17,23-epoxy-29-hydroxy-27-norlanosta-1,8-diene-3,15,24-trione; DG(8:0/16:0/0:0); and sugeonyl acetate, among others (R ≥ 0.6 or R ≤ −0.6, *p* < 0.05).

In the second functional community, g__Coriobacteriaceae_UCG-002 was significantly and positively correlated with the metabolites 3β-hydroxy-6β-angeloyloxy-7(11)-eremophilen-12,8β-olide; (3β,17α,23S)-17,23-epoxy-3,28,29-trihydroxy-27-norlanost-8-en-24-one; and salbutamol 4-O-sulfate; it was negatively correlated with D-erythro-sphingosine C-17 and sphingosine (R ≥ 0.6 or R ≤ −0.6, *p* < 0.05). The genus Faecalibaculum was significantly and positively correlated with (E)-2-(2-octenyl)cyclopentanone; (3S,4S,6R,7S)-1,10-bisaboladiene-3,4-diol; (±)-enterolactone; DG(8:0/16:0/0:0); and 2,4,5,7α-tetrahydro-1,4,4,7a-tetramethyl-1H-inden-2-ol (R ≥ 0.6 or R ≤ −0.6, *p* < 0.05). g__CAG-352 was significantly and positively correlated with the metabolites salbutamol 4-O-sulfate; 2,4,5,7α-tetrahydro-1,4,4,7a-tetramethyl-1H-inden-2-ol; 2,4,6-undecatrienal; glutamyltryptophan; and (17α,23S)-17,23-epoxy-29-hydroxy-27-norlanosta-1,8-diene-3,15,24-trione, among others; it was negatively correlated with the metabolites D-erythro-sphingosine C-17, sphingosine, spermidine, and simmondsin (R ≥ 0.6 or R ≤ −0.6, *p* < 0.05). Finally, members of the genus Lactobacillus were significantly and positively correlated with the metabolites (E)-2-(2-octenyl)cyclopentanone, (±)-enterolactone, and tetradecanedioic acid and negatively correlated with D-erythro-sphingosine C-17 and sphingosine (R ≥ 0.6 or R ≤ −0.6, *p* < 0.05).

### 2.10. Correlation Analysis between Ileal and Plasma Metabolites

Using Spearman’s correlation, we probed possible correlations between the ileal metabolites and the plasma metabolites exhibiting the top 50 VIP values in the respective metabolomic analyses. As shown in Figure 8B, the intestinal metabolites could be divided into two groups according to changes in correlation with plasma metabolites. The first functional group included 3β-hydroxycinnamolide; 5β-cholestane-3α,7α,24,26-tetrol; sphingosine; simmondsin; and spermidine; the second functional group included sugeonyl acetate; (E)-2-(2-octenyl)cyclopentanone; dodecanedioic acid; cholestane-3,7,12,25-tetrol-3-glucuronide; (17α,23S)-17,23-epoxy-29-hydroxy-27-norlanosta-1,8-diene-3,15,24-trione; 2,4,6-undecatrienal; trans-4-coumaric acid; and salbutamol 4-O-sulfate, among others.

In the first functional group, 3β-hydroxycinnamolide was significantly and positively correlated with the plasma metabolites lysoPC(20:4(8Z,11Z,14Z,17Z)), 4-hydroxybenzenesulfonic acid, and lysoPC(22:6(4Z,7Z,10Z,13Z,16Z,19Z)), and it was negatively correlated with hydrocortisone, lactupicrin, dolichin B, ercalcitriol, and trilostane, among others (R ≥ 0.6 or R ≤ −0.6, *p* < 0.05); 5β-cholestane-3α,7α,24,26-tetrol was significantly and positively correlated with the plasma metabolites 4-hydroxybenzenesulfonic acid and negatively correlated with ercalcitriol, hydrocortisone, lactupicrin, crustecdysone, trilostane, and others (R ≥ 0.6 or R ≤ −0.6, *p* < 0.05); sphingosine showed a significant positive correlation with the plasma metabolites 2-ethylacrylic acid, PC(20:4(8Z,11Z,14Z,17Z)/P-18:1(11Z)), and lysoPC(22:6(4Z,7Z,10Z,13Z,16Z,19Z)), and it showed a significant negative correlation with trans-dehydroandrosterone, dolichin B, stearidonic acid, ercalcitriol, and trilostane (R ≥ 0.6 or R ≤ −0.6, *p* < 0.05); simmondsin showed a significant negative correlation with ercalcitriol, trilostane, hydrocortisone, stearidonic acid, and dolichin B (R ≥ 0.6 or R ≤ −0.6, *p* < 0.05); finally, spermidine was significantly and positively correlated with the plasma metabolite 3-acetyl-2,7-naphthyridine and negatively correlated with dolichin B (R ≥ 0.6 or R ≤ −0.6, *p* < 0.05).

In the second functional group of ileal metabolites, sugeonyl acetate displayed a significant positive correlation with the plasma metabolites trilostane, hydrocortisone, ercalcitriol, aflatoxin B1 dialcohol, and physagulin C, and it displayed a negative correlation with the plasma metabolites 2-ethylacrylic acid; PC(20:4(8Z,11Z,14Z,17Z)/P−18:1(11Z)); and 3-acetyl-2,7-naphthyridine (R ≥ 0.6 or R ≤ −0.6, *p* < 0.05). (E)-2-(2-octenyl)cyclopentanone showed a significant positive correlation with the plasma metabolites 17-octadecene-9,11-diynoic acid; trilostane; dolichin B; hydrocortisone; and stearidonic acid, among others; it showed a significant negative correlation with the plasma metabolites lysoPC(22:6(4Z,7Z,10Z,13Z,16Z,19Z)), lysoPC(20:4(8Z,11Z,14Z,17Z)), PC(20:4(8Z,11Z,14Z,17Z)/P-18:1(11Z)), and N-fructosyl isoleucine (R ≥ 0.6 or R ≤ −0.6, *p* < 0.05); deoxycholic acid had a significant positive correlation with the plasma metabolites ganoderic acid H, hydrocortisone, ercalcitriol, medicagenic acid, and 5-aminosalicylic acid, among others, and it had a significant negative correlation with the plasma metabolites lysoPC(20:4(8Z,11Z,14Z,17Z)), lysoPC(22:6(4Z,7Z,10Z,13Z,16Z,19Z)), and 4-hydroxybenzenesulfonic acid (R ≥ 0.6 or R ≤ −0.6, *P* < 0.05); cholestane-3,7,12,25-tetrol-3-glucuronide was negatively correlated with the plasma metabolite 3-acetyl-2,7-naphthyridine (R ≥ 0.6 or R ≤ −0.6, *p* < 0.05); (17α,23S)-17,23-epoxy-29-hydroxy-27-norlanosta-1,8-diene-3,15,24-trione was significantly and positively correlated primarily with the plasma metabolites hydrocortisone, crustecdysone, ercalcitriol, trans-dehydroandrosterone, and floionolic acid, and it was negatively correlated with the plasma metabolites lysoPC(20:4(8Z,11Z,14Z,17Z)), 4-hydroxybenzenesulfonic acid, and lysoPC(22:6(4Z,7Z,10Z,13Z,16Z,19Z)) (R ≥ 0.6 or R ≤ −0.6, *p* < 0.05); 2,4,6-undecatrienal was significantly and positively correlated with the plasma metabolites hydrocortisone, 3-hydroxydodecanedioic acid, trilostane, lactupicrin, and dolichin B, among others, and it was negatively correlated with the plasma metabolites lysoPC(20:4(8Z,11Z,14Z,17Z)), PC(20:4(8Z,11Z,14Z,17Z)/P-18:1(11Z)), and lysoPC(22:6(4Z,7Z,10Z,13Z,16Z,19Z)) (R ≥ 0.6 or R ≤ −0.6, *p* < 0.05); trans-4-coumaric acid was significantly and positively correlated with the plasma metabolites crustecdysone, hydrocortisone, ercalcitriol, aflatoxin B1 dialcohol, and lactupicrin, and it was negatively correlated with the plasma metabolites N-fructosyl isoleucine, lysoPC(20:4(8Z,11Z,14Z,17Z)), PC(20:4(8Z,11Z,14Z,17Z)/P-18:1(11Z)), and lysoPC(22:6(4Z,7Z,10Z,13Z,16Z,19Z)) (R ≥ 0.6 or R ≤ −0.6, *p* < 0.05); finally, albutamol 4-O-sulfate had a significant positive correlation with the plasma metabolites hydrocortisone, stearidonic acid, dolichin B, crustecdysone, and trilostane, among others, and it had a significant negative correlation with the plasma metabolites 4-hydroxybenzenesulfonic acid and lysoPC(22:6(4Z,7Z,10Z,13Z,16Z,19Z)) (R ≥ 0.6 or R ≤ −0.6, *p* < 0.05).

### 2.11. Metabolomic KEGG Pathway Enrichment Analysis

**Figure 9:**
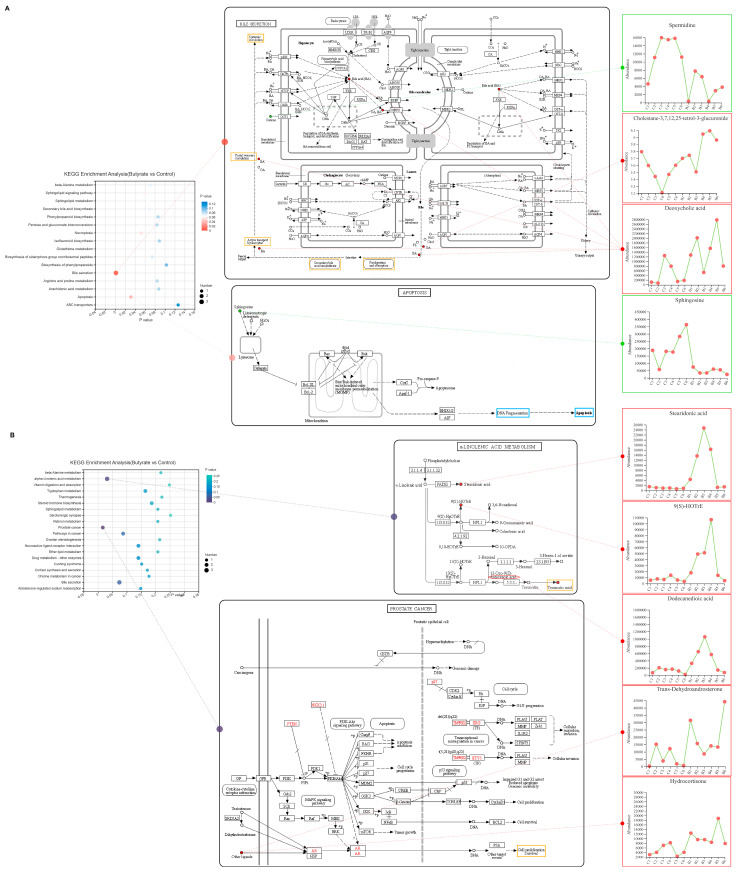
Metabolome KEGG pathway enrichment. (**A**) Ileal metabolome KEGG pathway enrichment. (**B**) Plasma metabolome KEGG pathway enrichment. Processes in yellow boxes are activated or upregulated; processes in blue boxes are inhibited or downregulated.

The KEGG pathway enrichment analysis (with Benjamini and Hochberg correction for multiple testing) of the ileal and plasma metabolite sets in the butyrate and control groups was performed to identify the major pathways associated with the differential metabolites in the metabolic sets using a hypergeometric distribution algorithm. We found that the ileal-dominant differential metabolites spermidine; cholestane-3,7,12,25-tetrol-3-glucuronide; and deoxycholic acid were significantly associated with the bile secretion pathway. We also found that sphingosine was significantly associated with the apoptosis pathway (Figure 9A, *p* < 0.05). In the bile acid secretion pathway, the increase in the abundance of cholestane-3,7,12,25-tetrol-3-glucuronide and deoxycholic acid in the intestinal lumen promoted the digestion and absorption of intestinal fat and the biosynthesis of secondary bile acids, activated active ileocyte transport, and finally enhanced the portal and systemic circulation of bile acids. In the apoptotic pathway, a decrease in sphingosine content in the intestine, which was regulated by a cascade in the pathway, eventually leads to a decrease in DNA fragmentation and a subsequent reduction in the levels of apoptosis. In plasma, the differential metabolites stearidonic acid, 9(S)-HOTrE, and dodecanedioic acid were significantly associated with the α-linolenic acid metabolism pathway, while trans-dehydroandrosterone and hydrocortisone were primarily associated with the prostate cancer pathway (Figure 9B, *p* < 0.05). In the α-linolenic acid pathway, the levels of stearidonic acid and 9(S)-HOTrE were increased, and the production of traumatic acid was increased by dodecanedioic acid. In the prostate cancer pathway, cell proliferation and survival were affected by trans-dehydroandrosterone and hydrocortisone, while there was no effect on other metabolic mechanisms in the pathway.

## 3. Discussion

Studies have increasingly focused on the production of short-chain fatty acid–butyric acids by anaerobic bacteria in the intestine. Sodium butyrate, a fatty acid salt, induces the functional maturation of intestinal epithelial cells by promoting their proliferation and inhibiting their apoptosis, thereby improving digestion and fostering intestinal mucosal development [27,28,29]. It has been reported that the supplementation of the diets of weaning piglets, calves, and lambs with an appropriate amount of butyrate can enhance their disease resistance, improve the intestinal microbiome, and increase growth performance [30,31,32]. In this study, we found that, in the weaned rabbits, dietary butyrate supplementation significantly increased the average daily feed intake and the average daily weight gain while reducing the rate of diarrhea. However, the beneficial effect of dietary butyrate on the weanling rabbits was greatest at 40–50 days of age and then subsequently decreased or even disappeared. Similar effects have been reported in piglets and lambs [33,34]. Nevertheless, the results of this and other studies have indicated that dietary butyrate exerts beneficial effects on rabbits and other mammals during the weaning period. Interestingly, in general, the adverse reactions induced by weaning in rabbits will gradually disappear after 60 days of age [35]. In summary, the 30–60-day-old stage of rabbits is the main period in which butyrate works, and in this study, we found the best beneficial effect during the 40–50-day-old period. Therefore, test samples collected during the 40–50-day-old test phase (at 50 days of age) were used for subsequent test analyses.

Many deleterious events can occur in the intestinal tract during early weaning, such as intestinal villi atrophy, crypt hyperplasia, intestinal inflammation, and increased intestinal permeability due to the disruption of tight junctions [8,36,37]. Several studies have shown that butyrate increases mucosal proliferation, improves epithelial cell differentiation and intestinal barrier function, and delays associated mucosal atrophy [38,39]. In our study, the H&E staining of intestinal sections and scanning electron microscopy results demonstrated that dietary butyrate promoted the growth of ileal villi, increased villi/crypt ratios, significantly reduced intestinal mucosal damage, and helped maintain the structural integrity of the intestinal mucosa. Tight junctions are key structures that determine the establishment and stability of the epithelial barrier by mediating intercellular adhesion and creating mechanical and charged barriers for the selective penetration of macromolecules and ions [40,41]. Occludins, claudins, and JAM are considered to be the major components of tight junctions, while ZO-1 is an adaptor protein that brings several tight junction components together and links them to the cytoskeleton [41,42]. As shown by our RT-qPCR results for the intestinal luminal surface mucosa, dietary butyrate significantly upregulated the expression of the claudin-1, claudin-2, occludin, *JAM3*, and *ZO-1* genes, which further explains the internal mechanism underlying how dietary butyrate helps maintain the integrity of the intestinal epithelial barrier.

Weaning disconnects young mammals from the passive immunity acquired from breast milk. The immune system of young animals is underdeveloped, frequently leading to inflammation of the digestive system, post-weaning diarrhea, and a reduction in free feeding [43,44,45]. In particular, when the first line of defense of the intestinal lumen is disrupted, intestinal permeability increases and allows toxins, bacteria, or feed-related antigens to cross the epithelium, which triggers active immunity and leads to inflammation and the release of pro-inflammatory factors [13]. The pro-inflammatory cytokines IL-1β, IL-6, and TNF-α play a central role in the cell-mediated immune response, while the NF-κB signaling pathway modulates the inflammatory response downstream of these pro-inflammatory factors [44,46]. In piglets and chicks, butyrate has been reported to exert its anti-inflammatory effects by reducing the expression of pro-inflammatory factors such as IL-1β, IL-6, and TNF-α and by inhibiting NF-κB pathway activation [47,48]. Consistent with the results of previous studies, we found that dietary butyrate downregulated the expression of IL-1β, IL-6, and TNF-α in the ileum, plasma, and hypothalamus to varying degrees and inhibited the activation of the NF-κB pathway in the ileum and hypothalamus. During inflammation, pro-inflammatory cytokines can enter the central nervous system and interact with cytokine networks in the brain, and they influence almost every aspect of brain function related to behavior, including neurotransmitter metabolism, neuroendocrine activity, mood, and the regulation of appetite [49]. The disruption of the epithelial barrier due to intestinal dysbiosis, infection, and injury results in the entry of bacteria and their metabolites into the bloodstream, which can promote systemic inflammation characterized by elevated plasma TNF-α, IL-6, and IL-1β levels. Blood–brain barrier permeability is subsequently increased, which allows for the entry of solutes and toxins into the brain [50]. IL-1β has been reported to have diverse effects on feeding behavior, including reducing gastric emptying and motility, as well as direct effects on the central nervous system [51]. CCK, PYY, and GLP-1 are neuroendocrine factors of intestinal origin that promote satiety, insulin secretion, and glucose handling, while ghrelin, a gastric hunger hormone, stimulates the appetite and is involved in the regulation of glucose homeostasis [52]. Signals from these peripheral gastrointestinal hormones are integrated into hypothalamic NPY feeding–stimulating neurons and POMC anorexigenic neurons that, in turn, modulate appetite and feeding behavior in animals [53]. Toll-like receptors in intestinal L cells have been reported to increase PYY expression by activating NF-κB signaling, while in macrophages, ghrelin inhibits the production of pro-inflammatory cytokines and has some anti-inflammatory effects [54,55]. In our study, rabbits in the butyrate group showed varying reductions in their CCK and PYY levels and increases in their ghrelin levels in the intestinal lumen and plasma. In the hypothalamus of the butyrate group, NPY expression was significantly upregulated, whereas that of POMC was suppressed. These results indicated that butyrate directly and indirectly reduces the release of intestinal pro-inflammatory factors through intrinsic anti-inflammatory properties and that dietary butyrate promotes the integrity of the intestinal epithelial barrier in weaned rabbits; this, in turn, alleviates weaning-induced intestinal epithelial barrier damage and inflammation in the animals. Furthermore, dietary butyrate suppressed the onset of hypothalamic inflammation and alleviated anorexic behavior in the weaning rabbits.

Overall, mammals maintain a mutually beneficial relationship with the intestinal prokaryotic community, a metabolically active entity that plays an important role in nutrition by degrading dietary substances that are indigestible by the host [56]. Young mammals are inoculated with bacteria as they pass through the birth canal, and their intestinal ecosystem undergoes profound changes as they are weaned from breast milk onto solid food [56,57]. Thus, the disruption of the intestinal microbiota structure at weaning can result in susceptibility to enterotoxigenic Escherichia coli, leading to post-weaning diarrhea in animals [58]. Butyrate, produced via the fermentation of undigested carbohydrates by bacteria in the intestine, plays an important role in intestinal health and has been used for the treatment of several inflammatory diseases [59,60,61]. Moreover, butyrate was reported to significantly alleviate the acute gastroenteritis caused by E. coli O157:H7, accelerate the clearance of the bacterium, and enhance resistance to E. coli O157:H7-induced disease [62]. In this study, we found that, although the alpha diversity of gut microorganisms was not significantly affected by dietary butyrate supplementation, beta diversity was significantly altered compared with that in the control group. Specifically, the abundance of g__Turicibacter, f__Peptostreptococcaceae, g__Terrisporobacter, g__Hungatella, and g__Jeotgalicoccus was significantly decreased in the gut of the weaning rabbits, whereas that of g__Coriobacteriaceae_UCG-002, g__Faecalibaculum, and g__CAG-352 was significantly increased. Regarding correlations with other bacteria, the gut microorganisms displaying a reduced abundance showed a significant positive correlation with g_Escherichia–Shigella, whereas those with an increased abundance showed a significant positive correlation with g_Lactobacillus. Abundant evidence supports that Shigella is the main cause of acute gastroenteritis in animals, while Lactobacillus can competitively exclude intestinal pathogens, produce antimicrobial compounds, modulate the host immune response, and maintain intestinal barrier integrity [63,64]. Studies have suggested that g__Turicibacter, found to be differentially dominant in the control group in our study, may be related to the intestinal barrier and host immune cells, and an increased abundance of g__Turicibacter may lead to a decreased expression of tight junction proteins. Less is known about the functions of f__Peptostreptococcaceae, the abundance of which is known to be increased in patients with colon cancer [65,66]. The genus Terrisporobacter is a close relative of Clostridium and is mainly associated with inflammatory bloodstream infections; g__Hungatella is also considered a pathogen and has been identified in patients with bacteremia and liver abscess [67,68]; g__Jeotgalicoccus belongs to the Staphylococcaceae family and is closely associated with enteritis; and Staphylococcus is a recognized causative agent of diarrhea, and its abundance is significantly increased in patients with digestive disorders [69]. In contrast, g__Coriobacteriaceae_UCG-002, found to be differentially dominant in the butyrate group in our study, can increase bile acid absorption, thereby influencing steroid and bile salt metabolism and reducing fecal steroid excretion [70]. Moreover, the bile salt hydrolase of g__Coriobacteriaceae_UCG-002 plays an important role in detoxification, and the abundance of g__Coriobacteriaceae_UCG-002 is reduced in the presence of inflammation [71]. g__Faecalibaculum is a potentially beneficial bacterium with anticancer properties [72]. However, little has been reported about the functions of g__CAG-352. In our study, we found that g__Terrisporobacter and g__Turicibacter, which were differentially dominant in the control group, may promote the release of intestinal inflammatory factors, induce inflammation, decrease ghrelin levels, reduce food intake, and, ultimately lead to diarrhea in rabbits. Similarly, g__Jeotgalicoccus and g__Hungatella were found to positively regulate intestinal pro-inflammatory factors, which may lead to damage to the intestinal epithelial barrier. However, some microorganisms that were differentially dominant in the butyrate group, such as g__Coriobacteriaceae_UCG-002, g__Faecalibaculum, and g__CAG-352, may have inhibited the release of intestinal pro-inflammatory factors while promoting intestinal epithelial barrier development and ghrelin release, thereby avoiding weaning-related anorexia in the rabbits.

The totality of metabolites produced in the intestine is referred to as the intestinal metabolome. As these metabolites and their small metabolic intermediates regulate intestinal immunometabolic homeostasis and are involved in host–microbe interactions, including reciprocity, symbiosis, and pathogenicity, the intestinal metabolome is among the strongest drivers of host–microbe interactions [73]. Intestinal metabolites, both from dietary and microbial sources, affect host energy homeostasis, fat storage, glucose metabolism, immune regulation, and endocrine function [74]. In this study, we found that dietary butyrate had a significant effect on the ileal metabolome. The differentially dominant metabolites that exerted a major bioactive function in the control group included simmondsin, sphingosine, spermidine, and 3β-hydroxycinnamolide; those in the butyrate group included 5β-cholestane-3α,7α,24,26-tetrol; cholestane-3,7,12,25-tetrol-3-glucuronide; deoxycholic acid; (E)-2-(2-octenyl)cyclopentanone; salbutamol 4-O-sulfate; trans-4-coumaric acid; sugeonyl acetate; (17α,23S)-17,23-epoxy-29-hydroxy-27-norlanosta-1,8-diene-3,15,24-trione; and 2,4,6-undecatrienal. Simmondsin, a differentially dominant metabolite in the control group, was reported to be a glycoside of jojoba meal that reduced food intake after oral administration [75]. Sphingosine is a metabolite of sphingolipids, which are important components of cell membranes. In addition, sphingosine is a negative regulator of cell proliferation that can inhibit cell growth and promote apoptosis [76]. Putrescine (polyamines) is synthesized by putrescine-encoding and putrescine-transferring enzymes containing putrescine, while spermidine is the main raw material for the synthesis of putrescine in the intestine [77]. Microbiota-derived polyamine metabolites were reported to exacerbate the damage to intestinal epithelial tight junctions and promote local and systemic inflammation [78]. In our study, we further found that the metabolites simmondsin, sphingosine, and spermidine enhanced the release of intestinal pro-inflammatory factors, downregulated the expression of intestinal epithelial barrier-related genes, reduced feed intake, and caused diarrhea in the weaned rabbits, among other negative effects. Relatively little is known about the functions of the metabolites 3β-hydroxycinnamolide and 5β-cholestane-3α,7α,24,26-tetrol, and we revealed important findings regarding these metabolites in the present study. Both metabolites showed significant positive correlations with the release of the pro-inflammatory factors IL-6 and TNF-α in the intestine, indicating that elevated levels of both are likely to induce enteritis, increase the diarrhea rate, and reduce body weight gain, the feed conversion rate, and feed intake in rabbits. Additionally, we identified a mutually reinforcing, positive relationship between differentially dominant microorganisms and differentially dominant metabolites in the ileum of the butyrate group.

Among the metabolites that were differential dominant in the butyrate group in this study, Fang et al. found that (E)-2-(2-octenyl)cyclopentanone was negatively correlated with inflammatory factors and harmful microorganisms [79]. In animals, bile acids, such as deoxycholic acid, promote proliferation in the intestinal mucosa [80]. Zhang et al. identified sugeonyl acetate as a putative active ingredient in Cyperi rhizoma with the potential for use in the treatment of cardiovascular disease and gastrointestinal disorders [81]. Salbutamol 4-O-sulfate is an inactive metabolite of albuterol that is primarily produced in the liver of animals. Its specific bioactive function is unknown [82]. Bile acids play a crucial role in lipid metabolism and their downregulation is indicative of impaired lipolysis, while a reduced abundance of cholestane-3,7,12,25-tetrol-3-glucuronide may imply an imbalance in bile acid metabolism [83]. Coumaric acid, an early product of the phenylpropanoid pathway, is weakly active on its own and is not directly involved in the systemic induction of antibacterial activity; however, its isomer trans-4-coumaric acid was reported to possess antifungal properties [84]. In the present study, we additionally found that deoxycholic acid, (E)-2-(2-octenyl)cyclopentanone, sugeonyl acetate, and salbutamol 4-O-sulfate can inhibit the release of intestinal pro-inflammatory factors and that cholestane-3,7,12,25-tetrol-3-glucuronide; (E)-2-(2-octenyl)cyclopentanone; sugeonyl acetate; and salbutamol 4-O-sulfate may aid in the maintenance of mucosal integrity by upregulating the expression of intestinal epithelial barrier-related genes. Meanwhile, salbutamol 4-O-sulfate was found to promote ghrelin secretion but to inhibit CCK and PYY secretion. No study to date has reported on the metabolites (17α,23S)-17,23-epoxy-29-hydroxy-27-norlanosta-1,8-diene-3,15,24-trione and 2,4,6-undecatrienal. Here, we identified the possible bioactive functions of these ileal metabolites in rabbits for the first time. Like the other dominant differential metabolites in the butyrate group, the functions of both metabolites may be associated with the inhibition of intestinal pro-inflammatory factor release, the upregulation of intestinal epithelial barrier-related gene expression, the promotion of gastrointestinal ghrelin release, and the inhibition of PYY and CCK synthesis. As expected, the relationship between the differentially dominant metabolites and the differentially dominant bacteria in the butyrate group was mutually reinforcing and positive; however, the relationship with the differentially dominant bacteria in the control group was mutually inhibitory and negative. The KEGG is a database resource for the systematic analysis of gene functions and for linking genomic information to functional information. We classified the metabolites in the metabolic set according to the pathways they participate in or the functions they perform using this database. In this study, we enriched by ileal metabolites to the bile acid secretion and apoptosis pathways. Bile acids are entirely synthesized from cholesterol in the liver and are subsequently released into the gastrointestinal tract after ingestion to help absorb nutrients, dietary fats, steroids, vitamins, and drugs [85]. Sphingolipids are ubiquitous components of cell membranes, and their metabolites ceramide, sphingosine, and sphingosine-1-phosphate have important physiological functions, including the regulation of cell growth and survival. Ceramide and sphingosine are associated with growth arrest and apoptosis, and their levels can be upregulated by a wide variety of stimuli, thereby promoting apoptosis [86]. Here, we found that dietary butyrate enhanced fat digestion and absorption in the intestine, promoted secondary bile acid biosynthesis, increased active ileocyte transport, and enhanced bile acid portal and systemic circulation through the combined action of the ileal metabolites spermidine, deoxycholic acid, and choline-3,7,12,25-tetrasaccharide. Meanwhile, dietary butyrate reduced the abundance of sphingosine in the ileum of the rabbits and, consequently, the occurrence of apoptosis.

Plasma represents a less invasive biomatrix that can reflect the dynamic changes occurring in the metabolome of the whole organism and is employed in most metabolomics studies [87]. Here, we found that dietary butyrate also exerted significant effects on the plasma metabolome. The metabolites that exerted major bioactive functions in the control group included lysoPC(22:6(4Z,7Z,10Z,13Z,16Z,19Z)), PC(20:4(8Z,11Z,14Z,17Z)/P-18:1(11Z)), lysoPC(20:4(8Z,11Z,14Z,17Z)), N-fructosyl isoleucine, 2-ethylacrylic acid, and 3-acetyl-2,7-naphthyridine. Those that exerted the main bioactive function in the butyrate group entirely differed from those of the control group and included trilostane, dolichin B, hydrocortisone, stearidonic acid, dodecanedioic acid, ercalcitriol, trans-dehydroandrosterone, and 9(S)-HOTrE. Glycerophospholipids are the major lipid components of cell membranes and have a wide range of biological functions, including cell differentiation, proliferation, and apoptosis. Lysophosphatidylcholines (lysoPCs) are formed by the hydrolysis of glycerophospholipids through the removal of one of the fatty acid moieties, and they are thought to be products of fatty acid oxidation that, in turn, affects the lipid content of tissues [88]. Increased oxidative stress disrupts lipid metabolism and prevents cell membrane biosynthesis [89]. Phosphatidylcholine has an important role in cell membranes; its dysregulated metabolism affects cell membrane synthesis and promotes cell membrane disruption, and it is a prominent feature of several cancers, including breast cancer [90]. Amino acid conversion in the liver mainly involves branched-chain and aromatic amino acids. Diao et al. found that, in diabetic patients, the levels of branched-chain amino acids and their derivatives, such as leucine, N-fructosyl-leucine, and N-fructosyl-isoleucine, were elevated in the liver, while that of N-fructosyl-valine was elevated in serum, indicative of an impaired amino acid metabolism [91]. In this study, we found that PC(20:4(8Z,11Z,14Z,17Z)/P-18:1(11Z)), lysoPC(22:6(4Z,7Z,10Z,13Z,16Z,19Z)), and plasma pro-inflammatory factors participated in a mutually reinforcing relationship. LysoPC(22:6(4Z,7Z,10Z,13Z,16Z,19Z)), PC(20:4(8Z,11Z,14Z,17Z)/P-18:1(11Z)), lysoPC(20:4(8Z,11Z,14Z,17Z)), and N-fructosyl isoleucine may have induced elevated the blood levels of PYY and CCK, promoted the expression of anorexigenic proteins in the hypothalamus, and suppressed the expression of appetite-promoting proteins. Relatively few studies have investigated the bioactive functions of the metabolites 3-acetyl-2,7-naphthyridine and 2-ethylacrylic acid in animals, and their effects in weaning rabbits are reported for the first time in this study. Like the bioactive functions of other dominant differential metabolites in the plasma of the control rabbits, these two metabolites may contribute to the release of pro-inflammatory factors by mutually inhibiting the expression of appetite-promotion-related proteins, enhancing the expression of appetite-suppressing proteins in the plasma and hypothalamus, and positively promoting the release of pro-inflammatory factors. Ultimately, in response to imbalances in body homeostasis during the weaning phase in rabbits, the dominant differential metabolites in the plasma of the control rabbits induced early-weaning anorexia and growth retardation, reduced feed conversion, and increased diarrhea rates.

The dominant differential metabolite in the butyrate group, trilostane, was shown to be a competitive inhibitor of the 3β-hydroxysteroid dehydrogenase isoenzyme system and is often used in animals to treat metabolic disorders due to pituitary-dependent hyperaldosteronism [92]. The dolichin protein has antifungal properties, endowing resistance to Fusarium oxysporum, Rhizoctonia solani, and Coprinus comatus, and it also inhibits HIV reverse transcriptase and α- and β-glucosidases (glycohydrolases that are associated with HIV infection). It has very low ribonuclease and cell-free translation-inhibitory activities [93]. Adrenocortical insufficiency typically results in impaired health and vitality, bone loss, and increased cardiovascular risk, and it is also associated with increased mortality. In the 1950s, hydrocortisone was introduced for the treatment of adrenocortical insufficiency to alleviate the associated metabolic disorders and ameliorate the quality of life of patients [94]. Whelan et al. reported that stearidonic acid has many biological functions in common with eicosapentaenoic acid, shares many of its beneficial effects on body metabolism, reduces inflammation, and prevents cancer. The authors proposed that stearidonic acid may be an alternative to eicosapentaenoic acid in health promotion and disease prevention [95]. Vitamin D2 is a fat-soluble vitamin that has very poor solubility and stability in aqueous solutions, and it can quickly and easily be oxidized and inactivated in a humid environment. Ergocalciferol, of plant origin, is metabolized by the body to ercalcitriol, an activated form of vitamin D2. Ergocalciferol is currently used for the prevention and treatment of vitamin D deficiency [96]. In the present study, we found that the metabolites trilostane, dolichin b, hydrocortisone, stearidonic acid, and ercalcitriol may have inhibitory effects on the release of pro-inflammatory factors in plasma. They could also have a reciprocal promotive effect with pro-appetite-related proteins in plasma and the hypothalamus, as well as a reciprocal inhibitory effect on the expression of anorexigenic proteins. In contrast to the bioactive functions of the dominant differential metabolites in the plasma of the control rabbits, the dominant differential metabolites in the butyrate group alleviated weaning-induced metabolic disorder, inhibited the occurrence of inflammation, enhanced feeding regulation, increased body weight gain and the feed conversion rate, and reduced the rate of diarrhea in the weaning rabbits. Interestingly, we found a strong correlation between the predominant differential metabolites in plasma and the predominant differential metabolites in the ileum, i.e., the predominant differential metabolites in the ileum and the predominant differential metabolites in plasma of the butyrate group displayed a positive mutual reinforcement. A similar effect was observed in the control group. However, the dominant differential metabolites in each group were in a negative relationship of mutual inhibition, and this could be extended to their respective relationships with gut microbes. Unlike the results of the KEGG pathway enrichment analysis involving ileal metabolites, differentially dominant plasma metabolites were associated with the α-linolenic acid metabolism pathway and the prostate cancer pathway. Many studies have found that α-linoleic acid can alleviate inflammation and tissue damage induced by inflammatory bowel disease and that appropriate doses of α-linoleic acid can exert protective effects against enteritis through the Th1/Th2/Th17 pathway. Both stearidonic acid and 9(S)-HOTrE in this pathway have anti-inflammatory bioactive functions; moreover, dodecanedioic acid exerts antioxidant and collagen synthesis-stimulating effects by promoting the synthesis of traumatic acid [95,97,98]. Regarding the prostate cancer pathway enrichment, no abnormal changes in other signaling factors that inhibit or promote carcinogenesis were observed, and the cell migratory ability was not affected. In this pathway, trans-dehydroandrosterone and hydrocortisone could activate androgen receptors, thereby enhancing cell proliferation without carcinogenic risk. These observations suggest that dietary butyrate can alleviate weaning-induced inflammation and tissue cell death through its effect on the plasma metabolome via a regulatory mechanism involving both the α-linolenic acid metabolism pathway and the prostate cancer pathway.

## 4. Material and Methods

### 4.1. Animals and Experimental Design

Based on a sodium butyrate addition experiment (the experimental design and results are presented in Supplementary Materials), we determined 0.3% sodium butyrate to be the optimal dose to add to the diet of the early-weaning rabbits. A total of 120 newly weaned male rabbits (30 days old) of similar body weight (803.50 ± 0.50 g) were randomly divided into a control group and a butyrate group. The rearing environment and rearing methods were the same as those described in previous studies (see Supplementary Materials). The rabbits in the control group were fed a basal diet (without sodium butyrate), and the butyrate group was fed the basal diet supplemented with 0.3% sodium butyrate for 30 days (the experimental design is shown in Figure S1).

### 4.2. Sample Collection

On days 10, 20, and 30 of the experiment, six rabbits in each group were randomly selected for sample collection. Blood was collected from the ear marginal vein into an anticoagulant-containing (sodium heparin) blood collection tube. These were then centrifuged at 3000 rpm for 10 min, following which the supernatant was transferred to a 1.5 mL centrifuge tube and stored at −80 °C. The rabbits were euthanized using the air embolization method. Samples of rabbit hypothalamus, ileum, and ileal contents were collected and rapidly stored in liquid nitrogen. An ileal tissue sample (3 mm^3^) was fixed in a 2.5% glutaraldehyde solution, while another (1.5 cm^3^) was fixed in a 4% paraformaldehyde solution.

### 4.3. Hematoxylin and Eosin (H&E) Staining and Scanning Electron Microscopy

Paraformaldehyde-fixed ileal samples were embedded in paraffin, sectioned, and submitted to H&E staining using conventional methods; the villus height and crypt depth were measured under a microscope.

A 3 mm^3^ ileal tissue sample was fixed in an electron microscope fixative (Servicebio, Wuhan, China). Immobilized samples were rinsed three times with 0.1 M PBS (Solarbio, Beijing, China), 15 min each wash, and then post-fixed in a 1% starvation acid solution at room temperature for 2 h, protected from light. After rinsing three times with 0.1 M PBS, 5 min each time, the tissues were sequentially immersed in 30%, 50%, 70%, 80%, 90%, 95%, and 100% alcohol, 15 min each time, and then immersed in isoamyl acetate for 15 min. Samples were then dried in a critical point drier and fixed on the ion sputterer sample stage with double-sided conductive carbon film adhesive and sprayed with gold for 30 s. Finally, the samples were observed and imaged under a scanning electron microscope.

### 4.4. Reverse-Transcription Quantitative PCR

Total RNA extraction and RT-qPCR were performed as previously described [99]. Exon–intron linker primers were designed using Primer 6.0 software (Primer-E Ltd., Plymouth, UK); the primer sequences are shown in Table S3. qPCR was performed following the method described in the Accurate Biology SYBR Green Premix Pro Taq HS qPCR Kit (Accurate Biology, Hunan, China). PCR data were analyzed using the 2-ΔΔCT method. Target gene mRNA levels were normalized to those of glyceraldehyde 3-phosphate dehydrogenase (GAPDH) and β-actin (ΔCT). The expression of GAPDH and β-actin was stable across treatments (*p* > 0.1).

### 4.5. Western Blot

Total proteins were extracted from ileal and hypothalamic tissue using a RIPA lysis buffer containing the protease inhibitor PMSF. After centrifugation at 12,000 rpm for 15 min at 4 °C, protein concentrations were measured using the BCA Protein Assay Kit (Thermo Fisher, Waltham, MA, USA). Total proteins were separated with 4–20% sodium dodecyl sulfate–polyacrylamide gel electrophoresis (ACE Biotechnology, Nanjing, China), transferred to polyvinylidene fluoride membranes (Millipore, Darmstadt, Germany), blocked for 1 h, and then incubated with primary antibody overnight at 4 °C. M5 Prestained Plus Protein Ladder (Mei5 Biotechnology, Beijing, China) was used as the protein marker. Mouse anti-β-actin monoclonal antibody (Bioss, Beijing, China) was used as a loading control. Immunoreactivity was detected using an enhanced chemiluminescence (ECL) kit (NCM Biotech, Suzhou, China) and visualized using the Fluor Chem M system. ImageJ software was used for quantitative analysis (NIH ImageJ system, Bethesda, MD, USA).

### 4.6. ELISA

Plasma and ileum contents were centrifuged at 3000 rpm for 30 s at 4 °C, and the supernatant was transferred to a 1.5 mL centrifuge tube. ELISAs were performed according to the instructions of the respective kits (IL-1β, TNF-α, IL-6, cholecystokinin (CCK), ghrelin, GLP-1, and peptide YY (PYY)) (Mlbio, Shanghai, China). Absorbance was measured at 450 nm using an enzyme marker, and the contents of IL-1β, TNF-α, IL-6, CCK, ghrelin, GLP-1, and PYY in the samples were calculated using the respective standard curves.

### 4.7. Immunofluorescence

The paraffin-embedding of sections and incubation with antibodies were performed as previously described [100]. Sections were gently shaken, sealed with an anti-fluorescence quenching agent (Thermo Fisher, Shanghai, China), and observed and imaged under a fluorescence microscope.

### 4.8. Ileum Microbial 16S rRNA Gene Sequencing

The primer pair 338F (5′-ACTCCTACGGGAGGCAGCAG-3′) and 806R (5′-GGACTACHVGGGTWTCTAAT-3′) was used for the amplification of the V3–V4 highly variable region of the bacterial 16S rRNA gene, which was performed in an ABI GeneAmp 9700 PCR Thermal Cycler (ABI, CA, USA). Sample processing and assays were performed as previously described [101]. The clustering of non-repetitive sequences (excluding single sequences) into operational taxonomic units (OTUs) was based on 97% similarity; chimeras were removed in the clustering process to obtain representative OUT sequences. Abundance bubble charts were used to analyze the species composition of the communities in the two groups. Principal coordinate analysis (PCoA) was used for the comparative analysis of the beta diversity in samples from both groups. Significant differences between the two groups were determined using the linear discriminant analysis of effect size (LEfSe) method. The FAPROTAX database was used for functional prediction and between-group difference testing.

### 4.9. Ileum and pl|Asma LC–MS-Based Untargeted Metabolomic Analysis

Samples were processed and measured according to the method described by Li et al. [102]. Metabolite samples from both groups were compared using partial least squares discriminant analysis (PLS–DA). Changes in the importance and expression of the different metabolites were assessed based on variable importance in projection (VIP) scores. A Kyoto Encyclopedia of Genes and Genomes (KEGG; http://www.genome.jp/kegg/; accessed on 9 December 2022) pathway enrichment analysis of the different metabolites was also undertaken.

### 4.10. Statistical Analyses

The analysis of OUT clustering, the evolutionary tree, and microbiome diversity was performed using Usearch (v.11; http://www.drive5.com/usearch/; accessed on 9 December 2022), Mega (v.7.0; https://www.megasoftware.net/; accessed on 9 December 2022), and Qiime (v.1.9.1; http://qiime.org/install/index.html; accessed on 9 December 2022) software, respectively. The KEGG pathway enrichment (kegg_v94.2), VIP scores, and orthogonal partial least squares discriminant analysis (OPLS–DA) of the metabolomics data were performed using the KEGG NETWORK database (Release, 2017-05-01) and R (ropls, v.1.6.2) software. Other data were analyzed using SPSS 22 software. The Student’s *t*-test was used to compare differences between the two groups (*p*-values < 0.05 were considered significant). Figures were prepared with Adobe Illustrator 2018 and GraphPad Prism 8 software.

## 5. Conclusions

In this study, we found that dietary butyrate promoted the transcription of tight junction-related genes in the intestinal epithelial barrier and altered the structural composition of intestinal microbes in weanling rabbits. The abundance of harmful bacteria (g__Turicibacter, f__Peptostreptococcaceae, g__Terrisporobacter, g__Hungatella, and g__Jeotgalicoccus) was reduced while that of beneficial bacteria (g__Coriobacteriaceae_UCG-002, g__Faecalibaculum, and g__CAG-352) was increased. The abundance of beneficial metabolites in the intestinal metabolome ((E)-2-(2-octenyl)cyclopentanone; deoxycholic acid; sugeonyl acetate; salbutamol 4-O-sulfate; cholestane-3,7,12,25-tetrol-3-glucuronide; (17α,23S)-17,23-epoxy-29-hydroxy-27-norlanosta-1,8-diene-3,15,24-trione; and 2,4,6-undecatrienal) and the plasma metabolome (trilostane, dolichin B, hydrocortisone, stearidonic acid, dodecanedioic acid, ercalcitriol, trans-dehydroandrosterone, and 9(S)-HOTrE) was increased in the butyrate group relative to that of the control group; meanwhile, the abundance of unbeneficial metabolites (intestinal metabolites: simmondsin, sphingosine, spermidine, 3β-hydroxycinnamolide, and 5β-cholestane-3α,7α,24,26-tetrol; plasma metabolites: lysoPC (22:6(4Z,7Z,10Z,13Z,16Z,19Z)), PC(20:4(8Z,11Z,14Z,17Z)/P-18:1(11Z)), lysoPC(20:4(8Z,11Z,14Z,17Z)), N-fructosyl isoleucine, 3-acetyl-2,7-naphthyridine, and 2-ethylacrylic acid) was decreased. The microbiome and metabolome had a positive and mutually reinforcing relationship mediated by dietary butyrate. Moreover, dietary butyrate promoted both fat digestion and absorption in the intestine and bile acid circulation by modulating the bile acid secretion pathway, enhanced anti-inflammatory and antioxidant functions via the α-linolenic acid pathway, reduced apoptosis via the apoptosis pathway, and promoted cell division and survival by modulating mechanisms related to the prostate cancer pathway. The beneficial effects exerted by dietary butyrate included the protection and maintenance of the intestinal epithelial barrier, the suppression of inflammation, and the mitigation of post-weaning anorexia in rabbits. In this study, we elucidated the mechanism of action associated with the effects of butyrate on the microbial–gut–brain axis for the first time and found that it involves a multidimensional regulatory process that effectively alleviates metabolic disorders, post-weaning anorexia, and early-weaning-related growth retardation. Our findings provide a rationale for the application of butyrate in animal production and, consequently, in the promotion of human health.

## Data Availability

The data that support the findings of this study are available from the corresponding author upon reasonable request. The raw omics data can be obtained by clicking the following link (http://www.ncbi.nlm.nih.gov/bioproject/896893; accessed on 9 December 2022).

## Supplementary Materials

The following supporting information can be downloaded at: https://www.mdpi.com/article/10.3390/ijms24021787/s1. References [103,104] are cited in the supplementary materials.
