# Supplementary material for: Combined Omics Analysis Further Unveils the Specific Role of Butyrate in Promoting Growth in Early-Weaning Animals
Source: Int J Mol Sci. 2023 Jan 16;24(2):1787. doi: 10.3390/ijms24021787 (PMC9864007; doi:10.3390/ijms24021787)
Supplement: Supplementary file 1 [file ijms-24-01787-s001.zip › ijms-2121887-supplementary.pdf]

International Journal of
Molecular Sciences

MDPI

Supplementary Materials

Combined Omics Analysis Further Unveils the Specific Role of Butyrate in Promoting Growth in Early Weaning Animals

Bin Zhang [1], Mengqi Liu [1], Zhengkai Yue [1], Xiaoyang Chen [1,2,3], Chenyang Li [1,2,4], Lei Liu [1,*], and Fuchang Li [1,*]

1 Key Laboratory of Efficient Utilization of Non-grain Feed Resources (Co-construction by Ministry and Province), Ministry of Agriculture and Rural Affairs, Shandong Provincial Key Laboratory of Animal Biotechnology and Disease Control and Prevention, Department of Animal Science, Shandong Agricultural University, Taian, Shandong 271018, China
2 State Key Laboratory of Animal Nutrition, Institute of Animal Science, Chinese Academy of Agricultural Sciences, Beijing 100193, China
3 College of Animal Science and Technology, Northwest A&F University, Shanxi 712100, China
4 College of Animal Science and Technology, China Agricultural University, Beijing 100083, China
* Correspondence: leiliu@sdau.edu.cn; chlf@sdau.edu.cn

Experimental Design

240 freshly weaned male rabbits (30 days old) of similar body weight were randomly divided into eight groups. In each group of 30 rabbits, 2 rabbits were kept in a 60 x 40 x 40 cm cage. The temperature of the rabbit house was approximately 23° C, with natural ventilation and light. Rabbits were allowed to eat and drink freely. According to our previous study [1], the eight experimental diets were supplemented with 0%, 0.05%, 0.10%, 0.30%, 0.50%, 0.70%, 0.90%, and 1.10% butyric acid to the basal diet (see Table S1 for diet composition and nutrient levels). The test basal diets were formulated according to NRC recommendations (1977) and Nutrition of Rabbit [2]. At the end of the experiment after 20 days, the average daily feed intake, average daily gain and diarrhea rate of rabbits were counted.

Results

As shown in Table S2, average daily feed intake and average daily gain were greatest when dietary butyric acid was 0.30%, while diarrhea rate was significantly lower ($p < 0.05$). This indicated that the addition of 0.30% butyrate to the rabbit diet was most appropriate.

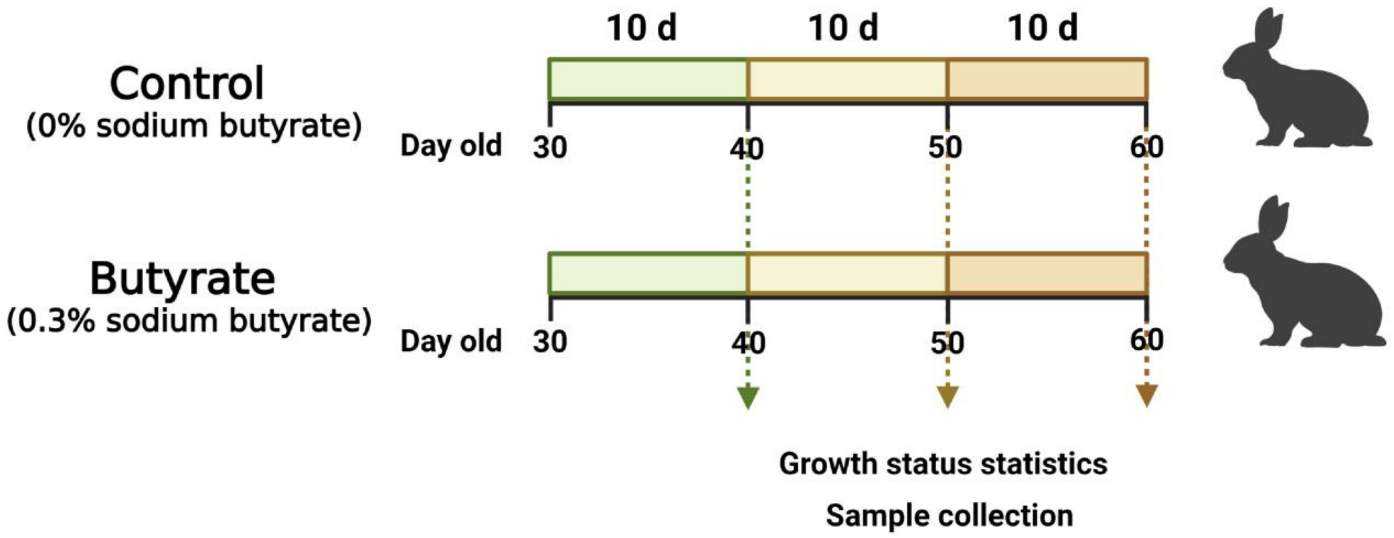

Figure S1. Experimental design.

Table S1. Composition and nutrient levels of basic diet (air-dry basis).

Ingredients	Content (%)	Nutrient levels[2]	Content
Corn	14	DE(MJ/kg)	9.94
Soybean meal	12	DM (%)	91.33
Wheat bran	13	CP (%)	15.67
Alfalfa meal	6.5	Ash (%)	7.46
Rice bran	15	EE (%)	4.12
Wheat middlings	8	CF (%)	15.04
Soybean husk powder	15	Ca (%)	0.86
Germ meal	3.5	P (%)	0.55
Bean straw powder	9		
Premix[1]	4		
Total	100		

[1] Table The premix provided the following per kg of diets: VA 12 000 IU, VD 900 IU, VE 50 mg, VK3 1 mg, VB1 1 mg, VB2 3 mg, VB6 1 mg, VB12 10 μg, choline chloride 500 mg, Fe (as ferrous sulfate) 100 mg, Zn (as Zinc) 50 mg, Se (as Selenium) 0.05 mg, I (as Iodine) 0.6 mg, Lys 1500 mg, Met 1,500 mg. The rest was miscellaneous meal carrier complement.

[2] Nutrient levels were measured values. DE=Digestible Energy; DM=Dry Matter; CP=Crude Protein; EE=Ether extract; CF=Crude Fiber; Ca=Calcium; P=Phosphorus.

Table S2. The growth condition of rabbits (n = 30).

Item	0%	0.05%	0.10%	0.30%	0.50%	0.70%	0.90%	1.10%	SEM	P-values
ADFI (g)	127.72^{ab}	128.90^{ab}	136.22^{ab}	141.98^{a}	137.49^{ab}	135.58^{ab}	126.11^{b}	124.44^{b}	4.39	0.1268
ADG (g)	44.64^{c}	50.69^{bc}	55.71^{ab}	59.24^{a}	55.22^{ab}	48.60^{bc}	44.29^{c}	47.97^{bc}	2.57	0.0007
Diarrhea rate (%)	23.25^{a}	18.22^{ab}	14.77^{ab}	9.85^{b}	14.95^{ab}	15.17^{ab}	16.78^{ab}	18.01^{ab}	3.31	0.2727

Abbreviations: ADFI = average daily feed intake; ADG = average daily gain.
Means without a common lowercase superscript letter in a row are different in $p < 0.05$.

Table S3. Gene-specific primers used for the analysis of rabbits gene expression.

Gene	Genebank accession number	Primers sequences(5′→3′)	Product size (bp)
β-actin	NM_001101683.1	F: CGCAGAAACGAGACGAGATT<br>R: GCAGAACTTTGGGGACTTTG	168
Claudin-1	NM_001089316.1	F: CAGCATGGTATGGCAACAGAA<br>R: TCCGAGGACAAGAACAGCAA	156
Claudin-2	XM_008272846.2	F: AGTGGCGATAGCAGGTGGAGTC<br>R: AGTGGTGAGTAGAAGTCCCGAAGG	105
GAPDH	NM_001082253	F: TGCCACCCACTCCTCTACCTTCG<br>R: CCGGTGGTTTGAGGGCTCTTACT	163
JAM2	XM_017346699.1	F: ATATCGCAGGTGTCCTGGAA<br>R: GAGCATAGCACACGCCAAG	122
JAM3	XM_008248363.2	F: TGTGAGGAGCAGGAGATGGA<br>R: GCCACGTCTGTATGCACAGC	123
Occludin	XM_017344772.1	F: CACGCTTGCCTGGGACAGAAC<br>R: CGTAGCCGTAACCATAGCCATAGC	116
ZO-1	XM_017348360.1	F: CCTCCGCTCATACCTTCCTCTCAG<br>R: GGTCCTCGTCCTCCTCGGTTAG	100

Abbreviations: GAPDH: Gyceraldehyde-3-phosphate dehydrogenase; JAM2/3: Junctional adhesion molecule2/3; *ZO-1*: Zonula occludens1

References

1. Li, C.; Chen, X.; Zhang, B.; Liu, L.; Li, F. Sodium butyrate improved intestinal barrier in rabbits. *Ital J Anim Sci* **2020**, 19, 1482-1492.
2. Blas, C.D.; Wiseman, J. Nutrition of the rabbit. *Nutrition of the rabbit* **2010**, (Ed. 2).
